# Pro‐migratory and TGF‐β‐activating functions of αvβ6 integrin in pancreatic cancer are differentially regulated via an Eps8‐dependent GTPase switch

**DOI:** 10.1002/path.4923

**Published:** 2017-08-07

**Authors:** Jo Tod, Christopher J Hanley, Mark R Morgan, Marta Rucka, Toby Mellows, Maria‐Antoinette Lopez, Philip Kiely, Karwan A Moutasim, Steven J Frampton, Durgagauri Sabnis, David R Fine, Colin Johnson, John F Marshall, Giorgio Scita, Veronika Jenei, Gareth J Thomas

**Affiliations:** ^1^ Cancer Sciences Unit, Faculty of Medicine University of Southampton, Tremona Road Southampton UK; ^2^ Institute of Translational Medicine University of Liverpool, Crown Street Liverpool UK; ^3^ Clinical and Experimental Sciences, Faculty of Medicine University of Southampton, Tremona Road Southampton UK; ^4^ Barts Cancer Institute, Barts and The London School of Medicine and Dentistry Queen Mary University of London, Charterhouse Square London UK; ^5^ IFOM FOM Foundation Institute FIRC of Molecular Oncology and University of Milan, School of Medicine, Department of Oncology and Hemato‐Oncology‐DIPO, Via Adamello Milan Italy

**Keywords:** αvβ6, Eps8, TGF‐β1, motility, Rac1, Rho

## Abstract

The integrin αvβ6 is up‐regulated in numerous carcinomas, where expression commonly correlates with poor prognosis. αvβ6 promotes tumour invasion, partly through regulation of proteases and cell migration, and is also the principal mechanism by which epithelial cells activate TGF‐β1; this latter function complicates therapeutic targeting of αvβ6, since TGF‐β1 has both tumour‐promoting and ‐suppressive effects. It is unclear how these different αvβ6 functions are linked; both require actin cytoskeletal reorganization, and it is suggested that tractive forces generated during cell migration activate TGF‐β1 by exerting mechanical tension on the ECM‐bound latent complex. We examined the functional relationship between cell invasion and TGF‐β1 activation in pancreatic ductal adenocarcinoma (PDAC) cells, and confirmed that both processes are αvβ6‐dependent. Surprisingly, we found that cellular functions could be biased towards either motility or TGF‐β1 activation depending on the presence or absence of epidermal growth factor receptor pathway substrate 8 (Eps8), a regulator of actin remodelling, endocytosis, and GTPase activation. Similar to αvβ6, we found that Eps8 was up‐regulated in >70% of PDACs. In complex with Abi1/Sos1, Eps8 regulated αvβ6‐dependent cell migration through activation of Rac1. Down‐regulation of Eps8, Sos1 or Rac1 suppressed cell movement, while simultaneously increasing αvβ6‐dependent TGF‐β1 activation. This latter effect was modulated through increased cell tension, regulated by Rho activation. Thus, the Eps8/Abi1/Sos1 tricomplex acts as a key molecular switch altering the balance between Rac1 and Rho activation; its presence or absence in PDAC cells modulates αvβ6‐dependent functions, resulting in a pro‐migratory (Rac1‐dependent) or a pro‐TGF‐β1 activation (Rho‐dependent) functional phenotype, respectively. © 2017 The Authors. *The Journal of Pathology* published by John Wiley & Sons Ltd on behalf of Pathological Society of Great Britain and Ireland.

## Introduction

The epithelial‐specific integrin αvβ6 is not expressed in healthy tissue but is up‐regulated during epithelial remodelling, where it interacts with several ligands including fibronectin, tenascin, vitronectin, and the latency‐associated peptide of TGF‐β1 (LAP) and TGF‐β3 1, 2, 3. αvβ6 is up‐regulated in numerous carcinomas, including pancreatic ductal adenocarcinoma (PDAC 4, 5, 6, 7), and a high level of expression is prognostic in several tumour types, including colorectal, lung, and breast carcinomas 6, 8, 9. Consistent with this, αvβ6 promotes tumour cell invasion and metastasis, partly by regulating multiple proteases [MMP‐9, MMP‐2, MMP‐13, and urokinase‐type plasminogen activator (uPA) [5,10]]. αvβ6 is also the principal epithelial activator of TGF‐β1 3, 11, interacting with its latency‐associated peptide to induce a conformational change in the latent complex to expose the active cytokine 12, 13. The potential for the therapeutic targeting of αvβ6 is complicated by its role in TGF‐β1 activation. While TGF‐β1 has numerous well‐described pro‐tumourigenic effects, including promoting tumour cell epithelial‐to‐mesenchymal transition (EMT) 6, immune suppression, and stromal myofibroblast differentiation 14, its role as a tumour suppressor in the stages of early carcinogenesis has raised the possibility that targeting the integrin in the wrong setting might act to promote, rather than suppress, tumour progression. Notably, inhibiting αvβ6 in a transgenic model of PDAC has been shown previously to promote early and late disease stages in the presence of wild‐type SMAD4 15.

Several studies have emphasized the importance of cell‐mediated mechanical force application in αvβ6‐dependent activation of TGF‐β1, whereby actomyosin‐dependent tractive and tensile forces are applied, via αvβ6, to ECM‐associated LAP 3, 11, 12, 13. Reorganization of the actin cytoskeleton is integral to cell migration, with tension transmitted to the ECM through integrin‐containing adhesion complexes at the cell surface; this is mediated through Rho GTPases (Rho, Rac, and Cdc42 16). Notably, epidermal growth factor receptor pathway substrate 8 (Eps8) has been shown to control actin remodelling both directly through actin capping and bundling and indirectly through activation of Rac1 17, 18, 19. Its multiple binding partners, including actin, Abi1, Sos1, RN‐Tre, IRSp53, palladin, F‐actin, and certain integrin β subunits (β1, β3, β5), indicate that Eps8 sits at the heart of a complex system regulating actin reorganization 17, 18, 19, 20, 21. Moreover, Eps8 up‐regulation in several cancers, including PDAC, has been shown to promote a pro‐migratory phenotype 22, 23, 24.

In this study, we examined the relationship between αvβ6‐dependent cell invasion and TGF‐β1 activation. Using PDAC (a cancer type with high levels of αvβ6 expression) as a model, we confirmed that both processes are primarily regulated by αvβ6. Surprisingly, we found that motility and TGF‐β1 activation have an inverse relationship, and that function can be biased towards either process depending on the presence or absence of Eps8, respectively. We found that Eps8, when in complex with Abi1/Sos1, promotes Rac1 activation, increasing cell migration and invasion; targeted siRNA knockdown of Eps8, Sos1 or Rac1 suppressed cell movement, but conversely, increased αvβ6‐dependent activation of TGF‐β1. This latter effect resulted from increased cell tension and was driven by Rho activation. The loss of motility following Rac1 or Eps8 knockdown was restored if Rho was also inhibited. Thus, the Eps8/Abi1/Sos1 tricomplex is a key regulator of αvβ6‐dependent tumour cell function, acting as a molecular switch that alters the balance between Rac1 and Rho activation, and biasing function towards a pro‐migratory (Rac1‐dependent) or pro‐TGF‐β1 activation (Rho‐dependent) phenotype, respectively.

## Materials and methods

### Antibodies and reagents

The antibodies and siRNA sequences used are summarized in the supplementary material, Tables S1 25 and S2.

### Cell lines and culture

Capan1 and SW1990 cells were cultured in DMEM, BxPC3, and SU86.86 cells in RPMI; and Panc0403 cells in RPMI supplemented with 10 mm HEPES buffer, 1 mm sodium pyruvate, and 0.2 U/ml insulin from bovine pancreas. Media contained 10% fetal bovine serum and 2 mm l‐glutamine. The absence of *Mycoplasma* was regularly confirmed using PCR. DNA fingerprinting of the PDAC cell lines (Capan1, BxPC3, and Panc0403) was performed using the GenomeLab Human STR Primer Set (Beckman‐Coulter Inc, Brea, CA, USA), and the results were verified against the COSMIC cell line database, Wellcome Trust Sanger Institute. Primary pancreatic stellate cells (PPSCs) were obtained using normal pancreatic tissue from two patients undergoing pancreatic resection at University Hospital Southampton (UHS). Appropriate ethical approval and patient consent were in place (REC No 10/H0502/72).

### RNA interference

Cells were transfected with pre‐designed, validated, and optimized siRNA oligonucleotides (supplementary material, Table S2 and Figure S1) using Oligofectamine™ transfection reagent according to the manufacturer's protocol (Invitrogen, Carlsbad, CA, USA). Functional assays were performed at 24 (organotypic assays) or 48 h (Transwell®/TGF‐β activation) post‐transfection.

### Transwell® migration and invasion assays

Motility assays were performed using Transwell® migration inserts (8 μm pore size, polycarbonate membrane; Corning® Costar® Wiesbaden, Germany) as described previously 10. For migration assays, the underside of the inserts was coated with 0.5 μg/ml latency‐associated peptide of TGF‐β1 (LAP); for invasion assays, the top of each insert was coated with Matrigel (BD Biosciences, San Diego, CA, USA) diluted 1:1 with DMEM. Cells migrating to/invading the lower chamber were counted after overnight (migration) or 72 h incubation (invasion) using a CASY counter (Sharfe System GmbH, Reutlingen, Germany).

### Organotypic cultures

Organotypic cultures were prepared as described previously and contained 2.5 × 10^5^ HFFF2/PPSC cells 26. 2.5 × 10^5^ HFFF2/PPSC cells combined with 5 × 10^5^ PDAC cells were seeded on top of the gels. The medium was changed every 2 days, and the gels were bisected, fixed, and processed to paraffin wax on day 12. Cell invasion was quantified using ImageJ software.

### Immunohistochemistry of tissue microarrays (TMAs)

TMAs were constructed from archival paraffin‐embedded material at UHS. Pancreatic resection specimens were derived from 75 patients undergoing pancreatic resection for PDAC between 2005 and 2010. H&E‐stained slides were reviewed and triplicate 1 mm cylindrical cores selected from representative areas of each tumour block and arrayed onto a new recipient paraffin block using a tissue arrayer (Alphelys Minicore® 3, Plaisir, France). Appropriate ethical and institutional ethical approval was obtained (REC No 10/H0502/72). Tissue staining was scored, using the QuickScore method, as absent/weak or moderate/strong 27.

### Western blotting

Cells were lysed in NP40 lysis buffer (40 mm Tris–HCl, pH 7.4, 1% NP40, 5 mm EDTA, 5 mm EGTA, 50 mm NaCl, 5 mm NaF, and protease inhibitor cocktail). Lysates containing equal amounts of protein were electrophoresed in 6–15% SDS‐PAGE gels and electroblotted to PVDF membranes (Merck Millipore, Watford, UK) as described previously 28. The antibodies used throughout the study are listed in the supplementary material, Table S1 25.

### Modulation and determination of Rac1 activity

Rac1 activation assays were performed as described previously 29. 8 × 10^5^ cells were plated 24 h post‐transfection and allowed to settle for 6 h. Cells serum‐starved overnight were lysed on ice following 5 min stimulation with EGF (20 ng/ml). Cleared cell lysates were incubated with GST‐PAK‐CRIB beads for 1 h at 4 °C. Active/total Rac1 levels were analysed by western blotting.

Transfection with constitutively active EGFP‐tagged Rac1 [Rac1V12‐GFP (J Monypenny, GKT, London, UK)] or vector control (pEGFP‐C2; Invitrogen) was performed using Fugene HD transfection reagents (Promega, Madison, WI, USA) following the manufacturer's protocol.

### Determination of Rho activity

Active/total RhoA levels were measured in 2 × 10^6^ cells 48 h post‐transfection using the Rho G‐LISA activation assay kit and the Total RhoA ELISA kit (Cytoskeleton, Denver, CO, USA) following the manufacturer's protocol.

### Phalloidin staining of PDAC cells

PDAC cells (4 × 10^5^, 24 h post‐transfection) were plated on 13‐mm LAP‐coated coverslips and serum‐starved overnight. Immunofluorescence staining was performed using the actin cytoskeleton staining kit (Merck Milllipore) according to the manufacturer's instructions. Scoring of actin stress‐fibre formation was performed in randomly selected, fully spread cells, in a minimum of ten fields per condition. Mean fluorescence intensity (arbitrary units) was calculated using ImageJ.

### Traction force microscopy

Ultrathin polyacrylamide hydrogels, embedded with FluoSpheres® carboxylate‐modified 0.2 μm fluorescent (505/515) microspheres (Thermo Fisher Scientific, Waltham, MA, USA), were generated as described previously 30, 31, 32 with some modifications. Briefly, NaOH‐washed 14 mm glass‐bottomed dishes (MatTek, Ashland, MA, USA) were amino‐silanated with 0.5% 3‐aminopropyltrimethyoxysilane and incubated with glutaraldehyde for 30 min. 20 μl of FluoSpheres® suspension was added to 500 μl of 3% or 9% acrylamide/bis‐acrylamide (37.5:1) solutions. 6 μl of polyacrylamide FluoSpheres® suspension was immediately added to amino‐silanated surfaces, overlaid with acid‐washed 13 mm coverslips, and allowed to polymerize, to produce hydrogels with predicted elastic moduli of 400 Pa and 12.5 kPa. Hydrogels were treated with 0.2 mg/ml sulfo‐SANPAH (Thermo Fisher Scientific), photoactivated with 365 nm UV light, washed with 50 mm HEPES (pH 8.5), and coated with 0.5 μg/ml LAP.

Cells were plated on LAP‐coated hydrogels for more than 14 h, prior to imaging on a 3i Marianas spinning‐disk confocal microscope. For each condition, multipoint bright‐field and 488 nm image stacks (> 12 μm) were acquired for each position. After imaging, cells were lysed with 1% SDS and multipoint positions re‐imaged to obtain unstrained bead positions.

Image analysis was performed using Fiji software on *z*‐position‐aligned pre‐ and post‐lysis stacks. Force‐induced bead displacement and gel deformation were measured by particle image velocitometry (PIV) using the iterative PIV (advanced) plugin 30 by three‐pass interrogation (third pass parameters: PIV3 interrogation window size = 40 px; SW3 search window size = 80 px; VS3 vector spacing = 20 px). Traction forces and vectors were calculated by Fourier transform traction cytometry (FTTC) using the FTTC plugin, predicted gel elastic moduli, and a Poisson ratio of 0.5 30.

### Co‐culture experiments

HFFF2 cells were plated in an eight‐well Permanox‐coated chamber slide (Thermo Fisher Scientific, Waltham, MA, USA) at a density of 10^4^ cells per well. After 48 h, 10^4^ cancer cells per well were plated on top of the fibroblasts. After 72 h, cells were fixed with 4% formaldehyde. Stress‐fibres were visualized with α‐smooth muscle actin (αSMA; Dako, Ely, UK), and cancer cells with cytokeratin (Dako) antibody using a Zeiss Axiovert 200 fluorescence microscope [at 40× magnification using an Orca‐ER digital camera (Hamamatsu, Shizuoka, Japan)]. αSMA mean fluorescence intensity (arbitrary units) was calculated using ImageJ software.

### TGF‐β activation assay

TGF‐β1 activation was measured by co‐culture of mink lung epithelial cells (MLECs) stably expressing a TGF‐β1‐responsive luciferase reporter construct (5 × 10^4^ cells per well) with PDAC cells (12 × 10^4^ cells per well) in serum‐free conditions 28.

### Statistical analysis

For functional assays, differences between experimental groups were examined with Student's *t*‐tests (Prism 4, GraphPad software). Statistical significance was indicated in the following way: *p < 0.05, **p < 0.01, ***p < 0.001, ****p < 0.0001. Bars indicate the standard deviation (SD) unless otherwise stated. Differences between immunohistochemical staining were examined using Fisher's exact test.

## Results

### αvβ6 is overexpressed in PDAC and promotes tumour invasion and TGF‐β1 activation

**Figure 1:**
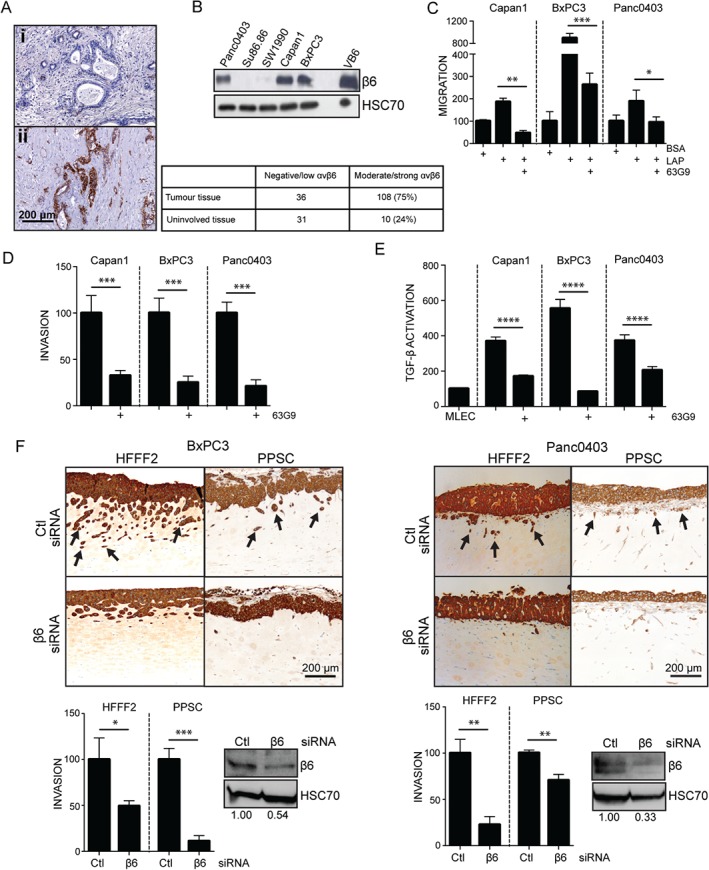
αvβ6 integrin is overexpressed in PDAC and promotes motility and TGF‐β activation. (A) Representative images of immunohistochemistry staining of αvβ6 in PDAC (ii; tumour) and surrounding (i; uninvolved) tissue. The table to the right shows the staining intensity using the QuickScore method. (B) Western blot showing β6 expression in PDAC cell lines. The human oral squamous cell carcinoma cell line VB6 was used as a positive control. Equal loading was confirmed by HSC70. (C) Transwell® migration towards LAP and (D) Matrigel invasion of αvβ6‐positive PDAC cells in the presence or absence of the αvβ6 blocking antibody 63G9. Diagrams represent the mean number of migrating/invading cells per well expressed as a % of Ctl (BSA) ± SD. n = 3 (migration); n = 4 (invasion); *p < 0.05; **p < 0.01; ***p < 0.001. (E) PDAC cells induce activation of TGF‐β1 in an MLEC TGF‐β activation assay, which is inhibited by 63G9. Diagram represents the mean relative light units expressed as a % of MLECs ± SD. n = 6; ****p < 0.0001. (F) Cytokeratin staining of organotypic gels showing invasion of non‐targeting (Ctl) or β6 siRNA‐transfected BxPC3 and Panc0403 cells in the presence of HFFF2 fibroblasts and primary pancreatic stellate cells (PPSCs). Representative images are shown. Arrowheads indicate invading tumour islands. Note the reduced invasion depth in organotypic cultures with β6 siRNA‐transfected cells. Diagrams showing the mean invasion depth of three independent sections analysed by ImageJ software expressed as a % of Ctl ± SD. n = 3; *p < 0.05; **p < 0.01; ***p < 0.001. Western blots confirm down‐regulation of β6. Equal loading was confirmed by HSC70. Numbers below the blots indicate the densitometry values measured using ImageJ normalized to HSC70 and expressed as a ratio to Ctl.

αvβ6 expression has been reported to be up‐regulated in PDAC 7. Our analysis of 75 tumours similarly showed significantly increased αvβ6 expression in PDAC tissue (75% moderate/strong expression) compared with surrounding tissue (24% moderate/strong expression) (Figure 1A). Five PDAC cell lines were screened for expression of the αv and β6 integrin subunits; three (Capan1, BxPC3, and Panc0403) showed high expression of both subunits (Figure 1B; supplementary material, Figure S2A) and were chosen for use in functional assays. All three cell lines showed αvβ6‐dependent migration and invasion, which were suppressed by an inhibitory αvβ6 antibody, 63G9 (Figure 1C, D; supplementary material, Figure S2B). We next performed TGF‐β1 activation assays and found that only cell lines with high αvβ6 expression showed robust activation of the cytokine; this was significantly reduced following αvβ6 blockade (Figure 1E; supplementary material, Figure S3). We then examined the role of αvβ6 in promoting collective tumour invasion in a 3D organotypic model using either human fetal foreskin fibroblasts (HFFF2) or primary pancreatic stellate cells (PPSCs) as the stromal component. BxPC3 and Panc0403 cells invaded well in the presence of either stromal cell type, with invasion significantly inhibited following β6 siRNA knockdown (Figure 1F). Capan1 cells do not invade in this model. These collective results confirm a central role for αvβ6 in tumour cell motility and TGF‐β1 activation in pancreatic cancer.

### Eps8 is overexpressed in PDAC, promotes αvβ6‐dependent PDAC motility, but suppresses αvβ6‐dependent TGF‐β1 activation

**Figure 2:**
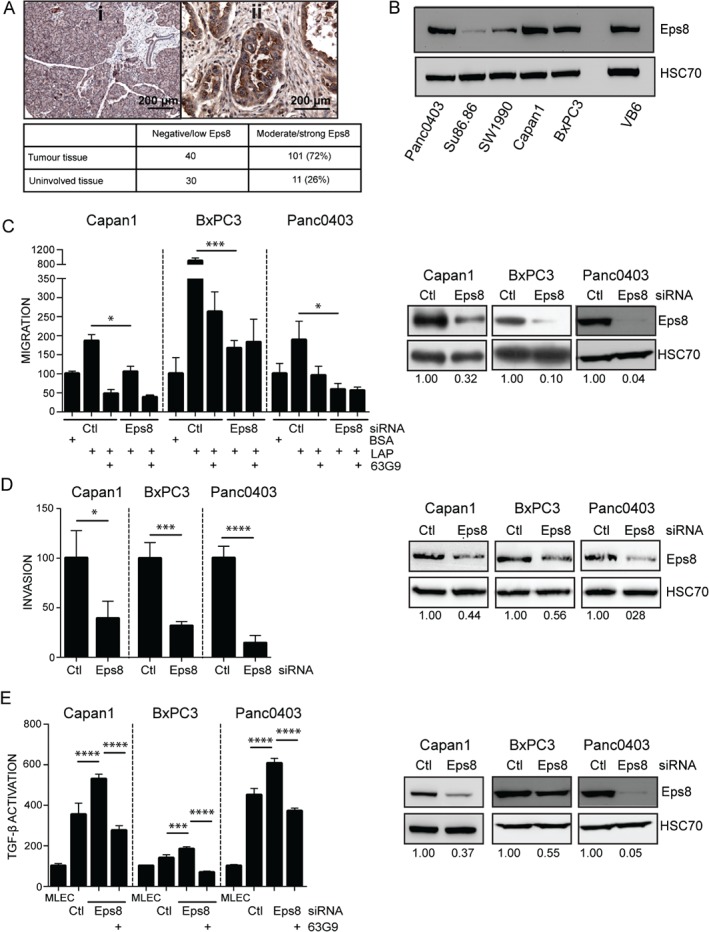
Eps8 is overexpressed in PDAC and promotes motility but inhibits TGF‐β activation. (A) Representative images of immunohistochemistry staining of Eps8 in PDAC (ii; tumour) and surrounding (i; uninvolved) tissue. The table shows the staining intensity using the QuickScore method. (B) Western blot showing Eps8 expression in five PDAC cell lines. The human oral squamous cell carcinoma cell line VB6 was used as a positive control. Equal loading was confirmed by HSC70. (C) Transwell® migration towards LAP and (D) Matrigel invasion of αvβ6‐positive PDAC cells following Eps8 knockdown in the presence or absence of the αvβ6 blocking antibody 63G9. Diagram represents the mean number of migrating/invading cells per well expressed as a % of Ctl (BSA) ± SD. n = 3 (migration); n = 4 (invasion); *p < 0.05; ***p < 0.001; ****p < 0.0001. (E) TGF‐β1 activation by PDAC cells following Eps8 knockdown measured by an MLEC TGF‐β activation assay, in the presence or absence of the αvβ6 blocking antibody 63G9. Diagram represents the mean relative light units expressed as a % of MLECs ± SD. n = 6; ***p < 0.001; ****p < 0.0001. Western blots in C–E confirm down‐regulation of Eps8. Equal loading was confirmed by HSC70. Numbers below the blots indicate the densitometry values measured using ImageJ normalized to HSC70 and expressed as a ratio to Ctl.

Similar to αvβ6, Eps8 has also been reported to be up‐regulated in several carcinomas 22, 23, 24, 29. We examined Eps8 expression in the cohort of 75 patients and found significant up‐regulation in PDAC tissue (72%) compared with surrounding pancreatic tissue (26%) (Figure 2A). Of the five PDAC cell lines examined, the three αvβ6‐positive lines expressed high levels of Eps8 (Figure 2B). We next investigated the role of Eps8 in αvβ6‐dependent migration, invasion, and TGF‐β activation. We found that Eps8 siRNA knockdown significantly inhibited both αvβ6‐dependent migration (Figure 2C; supplementary material, Figure S4A) and invasion (Figure 2D), whereas overexpression of Eps8–EGFP induced cell motility (supplementary material, Figure S5A). Unexpectedly, down‐regulation of Eps8 induced, rather than inhibited, αvβ6‐dependent TGF‐β1 activation (Figure 2E; supplementary material, Figure S4B). Consistent with this, overexpression of Eps8–EGFP suppressed activation of the cytokine (supplementary material, Figure S5B, C). These data suggest that αvβ6 regulates both PDAC motility and TGF‐β1 activation, and that the presence of Eps8 shifts the balance of these functions towards motility.

### Eps8 mediates αvβ6‐dependent functions via Rac1 activation

**Figure 3:**
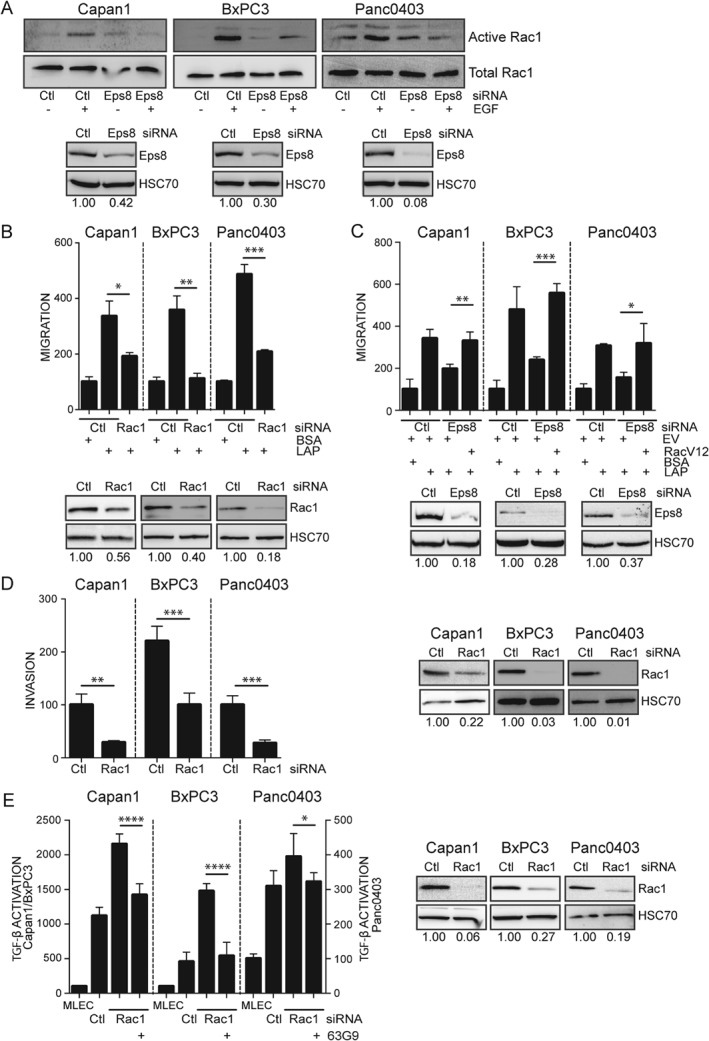
Eps8‐promoted Rac1 activation induces PDAC cell motility and inhibits TGF‐β activation. (A) Results of a GST pull‐down assay using GST‐PAK‐CRIB‐coated Sepharose beads showing EGF‐induced Rac1 activation in PDAC cells transfected with non‐targeting (Ctl) or Eps8 siRNA. Eps8 knockdown in the same lysates was confirmed on separate western blots. (B) Transwell® migration of PDAC cells transfected with non‐targeting (Ctl) or Eps8 siRNA towards LAP. Diagram represents the mean number of migrating cells per well expressed as a % of Ctl (BSA) ± SD. n = 3; *p < 0.05; **p < 0.01; ***p < 0.001. (C) Overexpression of the constitutively active mutant of Rac1 (RacV12) restores migration of PDAC cell lines compared with empty vector control (EV) following Eps8 knockdown. Diagram represents the mean number of migrating cells per well expressed as a % of EV (BSA) ± SD. n = 3; *p < 0.05; **p < 0.01; ***p < 0.001. (D) Matrigel invasion of PDAC cells was significantly inhibited by Rac1 knockdown. Diagram represents the mean number of invading cells per well expressed as a % of Ctl ± SD. n = 4; **p < 0.01; ***p < 0.001. (E) Rac1 knockdown induces activation of TGF‐β1 compared with non‐targeting (Ctl) siRNA‐transfected cells in an MLEC TGF‐β activation assay. Rac1‐induced TGF‐β1 activation was inhibited by the αvβ6 blocking antibody 63G9. Diagram represents the mean relative light units expressed as a % of MLECs ± SD (Capan1/BxPC3 plotted on left, Panc0403 plotted on right Y‐axis). n = 6; *p < 0.05; ****p < 0.0001. Western blots in B–E confirm down‐regulation of Rac1. Equal loading was confirmed by HSC70. Numbers below the blots indicate the densitometry values measured using ImageJ normalized to HSC70 and expressed as a ratio to Ctl.

Eps8 siRNA knockdown had no effect on αvβ6 levels/activation or cell adhesion (supplementary material, Figure S6), suggesting that Eps8‐dependent TGF‐β1 activation was not due to altered αvβ6 expression or function. Eps8 modulates the cytoskeleton via its actin‐binding and ‐capping activity, and through regulation of signalling pathways, including activation of the Rho GTPase, Rac1, when part of a tricomplex with Abi1 and Sos1 20. To determine whether Eps8 promotes Rac1 activation in PDAC cells, we performed Rac1 pull‐down assays using epidermal growth factor (EGF), a stimulus that amplifies αvβ6‐specific motility and Rac1 activation in these cell lines (supplementary material, Figure S7). Eps8 knockdown suppressed EGF‐dependent Rac1 activation (Figure 3A), which was similarly inhibited by knockdown of β6 (supplementary material, Figure S7C). Further investigation suggested involvement of the Eps8/Abi1/Sos1 tricomplex; we found up‐regulated expression of the Rac1 guanine nucleotide exchange factor, Sos1, in PDAC tissue and cell lines (supplementary material, Figure S8A, B), and Sos1 siRNA knockdown suppressed EGF‐induced Rac1 activation (supplementary material, Figure S8C). In motility assays, inhibition of Rac1 [siRNA, Figure 3B; Rac inhibitor NSC23766 (Merck Millipore), supplementary material, Figure S9A] significantly inhibited migration, and this was restored by expression of constitutively active Rac1V12 (Figure 3C). Inhibition of Sos1 also suppressed migration (siRNA, supplementary material, Figure S8D). Double knockdown of Eps8 and Rac1 did not produce a greater level of inhibition than that of the individual proteins (supplementary material, Figure S9B), suggesting that they function in the same pathway. PDAC cell invasion was also Rac1‐ and Sos1‐dependent (Figure 3D; supplementary material, Figure S8E).

Next, we examined the role of the Rac pathway in TGF‐β1 activation. Inhibition of Rac1 (siRNA, Figure 3E; NSC23766, supplementary material, Figure S9C) or Sos1 (siRNA, supplementary material, Figure S8F) resulted in a significant increase in αvβ6‐dependent TGF‐β1 activation. Similar to migration assays, double knockdown of Eps8 and Rac1 did not result in an additive effect (supplementary material, Figure S9D). These results provide evidence that Eps8, its binding partner Sos1, and their downstream effector Rac1 all promote αvβ6‐dependent PDAC motility, yet inhibit αvβ6‐dependent TGF‐β1 activation.

### Eps8 suppresses stress‐fibre formation and cell traction

**Figure 4:**
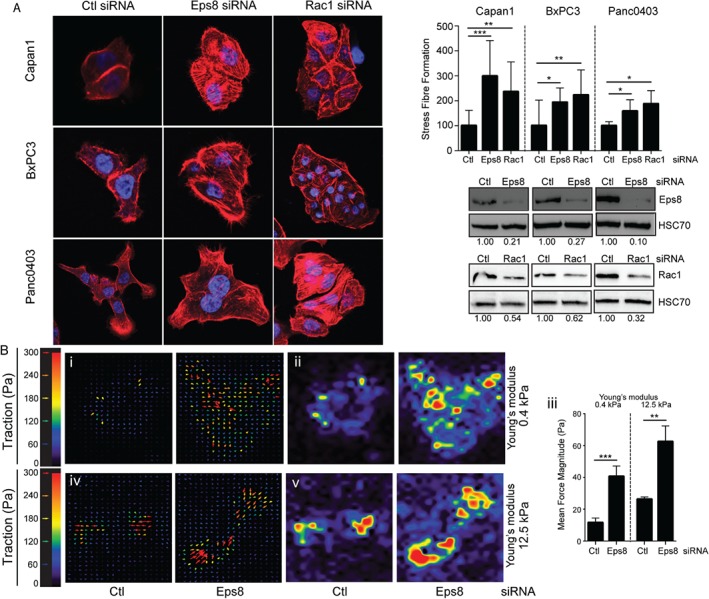
Inhibition of Eps8 promotes stress‐fibre formation and force application on LAP. (A) PDAC cells were transfected with either non‐targeting (Ctl) or Eps8/Rac1 siRNA, plated on 0.5 μg/ml LAP‐coated coverslips, and after an overnight incubation stained with phalloidin‐FITC (red) to visualize stress‐fibre formation. DAPI (blue) was used as a nuclear counterstain. Cells were visualized using ×100 optical zoom. Exposure of images was uniformly enhanced across images to aid better visibility. Actin stress‐fibre formation was quantified in randomly selected fully spread cells using ImageJ. Cells were selected as regions of interest (ROIs) and the phalloidin mean fluorescence intensity was quantified within the identified ROIs. Diagram represents the mean relative fluorescence intensity per field expressed as a % of Ctl ± SD. n = 10 fields per condition; *p < 0.05; **p < 0.01; ***p < 0.001. Western blots confirm down‐regulation of Eps8 or Rac1 following siRNA transfection. Equal loading was confirmed by HSC70. Numbers below the blots indicate the densitometry values measured using ImageJ normalized to HSC70 and expressed as a ratio of Ctl. (B) Cell‐mediated forces applied to 0.5 μg/ml LAP‐coated substrates, by BxPC3 cells transfected with non‐targeting (Ctl) or Eps8‐targeting (Eps8) siRNA, measured by traction force microscopy on hydrogels with predicted elastic moduli of 0.4 kPa (i, ii) and 12.5 kPa (iv, v). Data are represented as interrogation window force vectors (i and iv) and force magnitude maps (ii and v) for individual cells, and graphs (iii) showing the mean traction force magnitude per field ± SEM. n = 12–20 fields per condition; **p < 0.01; ***p < 0.001. Data show a representative experiment of three independent repeats.

αvβ6‐mediated TGF‐β1 activation is thought to require application of actomyosin‐dependent force on LAP to induce a conformational change in the TGF‐β1 latent complex 3, 11, 12, 13. Initially, we examined intracellular stress‐fibre formation as a surrogate measure of cytoskeletal tension. Eps8 or Rac1 knockdown led to a significant increase in stress‐fibre formation measured by mean fluorescence intensity of phalloidin staining (Figure 4A; supplementary material, Figure S10). We next examined whether Eps8 modulates tractive force using traction force microscopy to quantify cell‐mediated force applied to LAP substrates of differing rigidities. siRNA‐mediated knockdown of Eps8 in BxPC3 cells substantially increased the tractive forces, irrespective of substrate rigidity (Figure 4B). Together, these data suggest that Eps8‐dependent Rac1 activation inhibits TGF‐β1 activation through suppressing the force applied to the latent TGF‐β1 complex.

### Eps8 suppression promotes Rho activation and function

**Figure 5:**
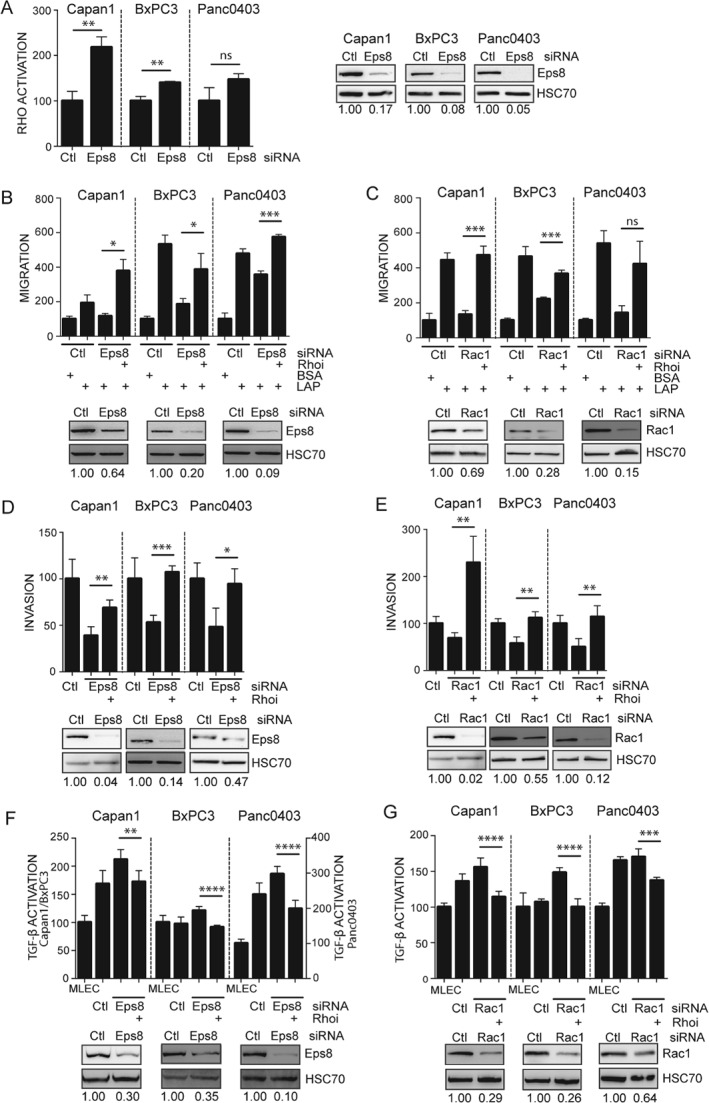
Eps8 and Rac1 regulate PDAC cell motility and TGF‐β activation by modulating RhoA. (A) PDAC cells were transfected with non‐targeting (Ctl) or Eps8‐targeting siRNA and RhoA activation was measured using a colorimetric G‐LISA assay 48 h post‐transfection. Diagrams represent the mean active RhoA levels normalized to total RhoA levels expressed as a % of Ctl ± SD. n = 3; **p < 0.01; ns = non‐significant. (B, C) Transwell® migration and (D, E) Matrigel invasion of PDAC cells transfected with non‐targeting (Ctl) or Eps8‐ (B, D) or Rac1‐ (C, E) targeting siRNA were measured following pretreatment with vehicle control or 0.5 μg/ml CT04 Rho inhibitor (Rhoi). Diagrams represent the mean number of migrating/invading cells per well expressed as a % of Ctl (BSA) ± SD. n = 3 (migration); n = 4 (invasion); *p < 0.05; **p < 0.01; ***p < 0.001; ns = non‐significant. (F, G) PDAC cells transfected with non‐targeting (Ctl) and Eps8‐ (F) or Rac1‐ (G) targeting siRNA were pretreated with vehicle control or 0.5 μg/ml CT04 Rho inhibitor (Rhoi), after which they were plated on top of MLEC cells in the absence of the inhibitor. Diagrams represent the mean relative light units expressed as a % of MLECs ± SD. n = 6; **p < 0.01; ***p < 0.001; ****p < 0.0001. Western blots confirm down‐regulation of Eps8 (A, B, D, F) and Rac1 (C, E, G). Equal loading was confirmed by HSC70. Numbers below the blots indicate the densitometry values measured using ImageJ normalized to HSC70 and expressed as a ratio to Ctl.

The role of Rho kinases in αvβ6‐dependent TGF‐β activation has previously been described 11, 33, 34, and Rac1 signalling has been shown to antagonize Rho activity directly 35, 36. We therefore hypothesized that Eps8/Rac1 suppression may promote RhoA activation, promoting increased cell tension. RhoA activation assays confirmed an increase in activation following down‐regulation of Eps8 (Figure 5A). To further examine the functional relationship between Rac1 and Rho signalling, we performed migration (Figure 5B, C) and invasion assays (Figure 5D, E) using the specific cell‐permeable exoenzyme C3 transferase‐based selective Rho inhibitor (CT04; Cytoskeleton) following siRNA knockdown of Eps8 or Rac1. Treatment of Eps8‐knockdown cells with this inhibitor led to reduced stress‐fibre formation, a readout of Rho activation (supplementary material, Figure S11). We also found that Rho inhibition following either Eps8 or Rac1 knockdown restored motility (Figures 5B–E). This suggests that reduced motility following Eps8/Rac1 suppression is the result, at least in part, of an increase in Rho activation and not simply loss of Rac activity. As anticipated, TGF‐β1 activation assays showed the reverse effect; the increase in αvβ6‐dependent TGF‐β1 activation seen following Eps8/Rac1 siRNA knockdown was suppressed by Rho inhibition (Figures 5F, G). Similarly, inhibition of the basal levels of Rho activation induced cell migration and inhibited TGF‐β1 activation (supplementary material, Figure S12). Our results show that the presence/absence of Eps8 signalling modulates different αvβ6‐dependent functions, through differential regulation of Rho GTPases; Eps8 promotes Rac1 activation and cell motility, yet suppression of this signalling pathway leads to an increase in Rho activation, which promotes TGF‐β1 activation.

### Differential effects of Eps8 on tumour–stromal interactions

**Figure 6:**
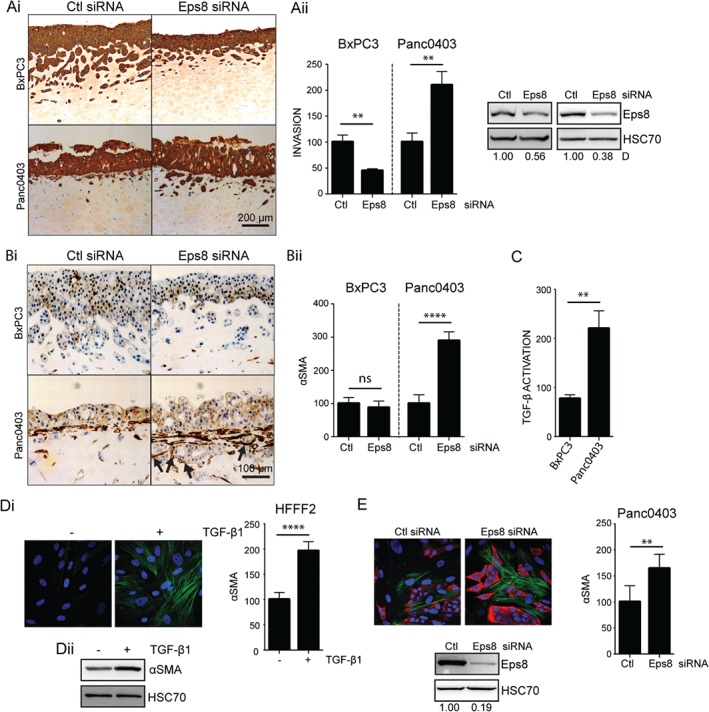
Differential activation of stromal cells by PDAC cell lines. (A, B) BxPC3 and Panc0403 cells were transfected with either non‐targeting (Ctl) or Eps8‐targeting siRNA and organotypic invasion assays were performed in the presence of HFFF2 fibroblasts over a period of 12 days. A representative image of a cytokeratin‐ (Ai) or the myofibroblast marker α‐smooth muscle actin‐stained (αSMA; Bi) section is shown. Arrowheads in Bi point at the interaction between invading tumour islands and activated fibroblasts. (Aii) Diagram showing the relative mean invasion depth of three independent sections analysed by ImageJ software expressed as a % of Ctl ± SD. **p < 0.01. (Bii) Diagram representing the αSMA‐positive area expressed as a % of Ctl ± SD analysed by the Trainable Weka segmentation plugin in the ImageJ software. n = 7; ****p < 0.0001; ns = non‐significant. Western blots confirm down‐regulation of Eps8. Equal loading was confirmed by HSC70. Numbers below the blots indicate the densitometry values measured using ImageJ normalized to HSC70 and expressed as a ratio of Ctl. (C) TGF‐β1 activation by BxPC3 and Panc0403 cells was measured by the MLEC TGF‐β activation assay and showed increased TGF‐β1 activation by Panc0403 cells. Diagram shows the results of seven independent experiments and represents the mean relative light units expressed as a % of MLECs ± SEM; **p < 0.01. (Di) HFFF2 fibroblasts were treated or not with hrTGF‐β1 for 48 h and αSMA‐positive stress‐fibres (green) were visualized by confocal microscopy. DAPI (blue) was used as a nuclear counterstain. Stress‐fibre formation was quantified in randomly selected fields using ImageJ. Diagram represents the mean relative fluorescence intensity per field expressed as a % of Ctl ± SD. n = 6 fields per condition; ****p < 0.0001. (Dii) TGF‐β1‐induced αSMA expression was confirmed using western blotting. (E) Panc0403 cells were transfected with non‐targeting (Ctl) or Eps8‐targeting siRNA and were plated on top of HFFF2 fibroblasts for 72 h. Stress‐fibre formation in the fibroblasts was detected by staining for αSMA (green); cancer cells were identified by cytokeratin staining (red) using confocal microscopy. DAPI (blue) was used as a nuclear counterstain. Stress‐fibre formation was quantified in randomly selected fields using ImageJ. Diagram represents the mean relative fluorescence intensity per field expressed as a % of Ctl ± SD. n = 6 fields per condition; **p < 0.01. Western blots in Aii and E confirm down‐regulation of Eps8. Equal loading was confirmed by HSC70. Numbers below the blots indicate the densitometry values measured using ImageJ normalized to HSC70 and expressed as a ratio of Ctl. Data show a representative experiment of at least three independent repeats.

In Transwell® assays, the pro‐migratory effect of Eps8 on cell invasion and migration was consistent across the different cell lines (Figure 2C, D) and independent of potential TGF‐β1‐dependent effects on cell movement. However, TGF‐β1 also promotes myofibroblast transdifferentiation of stromal cells 37; therefore, we examined the effect of Eps8 knockdown in 3D organotypic models containing fibroblasts or stellate cells. Surprisingly, we observed a differential effect of Eps8 on PDAC cell invasion; Eps8 suppression significantly reduced organotypic invasion of BxPC3 cells, but promoted invasion of Panc0403 cells (Figure 6A; supplementary material, Figure S13). A similar effect was observed following Rac1‐ and Sos1‐siRNA knockdown (supplementary material, Figure S14). TGF‐β1 activation can promote invasion through modulating stromal myofibroblast differentiation 38, and notably, immunohistochemical analysis highlighted a distinctive subepithelial layer of αSMA‐positive (activated) myofibroblasts in Panc0403 organotypic cultures (Figure 6B) that was not present in BxPC3 cultures. This desmoplastic reaction was enhanced following down‐regulation of Eps8 and revealed a clear interaction between αSMA‐positive fibroblasts and invading tumour islands. Treatment of HFFF2 fibroblasts with hrTGF‐β1 drives their differentiation from fibroblasts to contractile, αSMA‐positive myofibroblasts (Figure 6D); however, while both BxPC3 and Panc0403 cells activate TGF‐β1, direct comparison revealed that the levels of activation were significantly higher in Panc0403 cells (Figure 6C), possibly explaining the desmoplastic reaction in organotypic cultures with this cell line. Consistent with this, co‐culture experiments of HFFF2 fibroblasts with Eps8 knockdown Panc0403 cells showed significantly increased myofibroblast differentiation (Figure 6E). Thus, although Eps8 knockdown suppresses both Panc0403 and BxPC3 motility in Transwell® assays, the increased stromal response induced by the Panc0403 cells is sufficient to override this inhibition (which remains in the BxPC3 cells, due to their lack of ability to modulate the adjacent stroma).

## Discussion

Overexpression of αvβ6 is prognostic in various cancers 29, 38, 39; αvβ6 signalling promotes tumour invasion through different mechanisms, regulating protease expression and also activating the cytokine TGF‐β1, which promotes tumour cell EMT and stromal myofibroblast differentiation 40. Several studies suggest that αvβ6 targeting may be an attractive therapeutic option; targeting αvβ6 in pre‐clinical breast cancer models using a monoclonal antibody, 264RAD, either alone or in combination with the HER2 inhibitor trastuzumab, resulted in significant inhibition of disease progression 9, 41. However, targeting αvβ6 in human disease is complicated by its role in TGF‐β1 activation, which may act as a tumour suppressor through effects on cell cycle regulators 42. This has led to suggestions that αvβ6 should not be targeted in tumours retaining canonical TGF‐β1 signalling. For example, blockade of TGF‐β1 or αvβ6 in a genetically engineered KRAS PDAC mouse model was found to accelerate disease progression in Smad4‐expressing tumours 15. However, in tumours with homozygous deletion of SMAD4, the tumour‐suppressive effect of αvβ6 was lost 15. These data highlight the need to dissect the molecular mechanisms regulating functions of this integrin.

While αvβ6 has been shown to promote motility and TGF‐β1 activation in a number of tumour types, no study has examined yet how these functions are linked. Our previous work in oral squamous cell carcinoma showed that Eps8 regulates integrin‐mediated invasion 29, and we therefore examined more broadly the role of Eps8 in regulating αvβ6‐dependent functions. PDAC has been reported to overexpress both αvβ6 and Eps8 7, 23, and consistent with these studies, we found that around 70% of tumours expressed αvβ6 and Eps8 at moderate/high levels (Figures 1A and 2A). Similar to other cancer types, we found that αvβ6 promotes cancer cell motility and TGF‐β1 activation 37, 43, 44, with suppression of αvβ6 resulting in complete inhibition of these processes.

Eps8 has been shown previously to promote the migration of PDAC cells 23. We found that this motility‐promoting effect appears to be through modulating activation of Rac1 via the Rac1–GEF Sos1, as we found that suppression of Eps8, Sos1 or Rac1 independently inhibited PDAC migration/invasion. It is therefore probable that Eps8 regulates αvβ6‐dependent motility through the Eps8/Abi1/Sos1 tricomplex, although Eps8/Sos1 has also been shown to complex with CIIA, a protein associated with regulation of EMT and cell migration, to promote Rac1 activation independent of Abi1 45. Notably, the inhibitory effect of Eps8 knockdown on cell motility resulted from differential regulation of Rac1 and RhoA activation (Figure 5); reduced migration following Eps8/Rac1 inhibition could be restored either by expressing constitutively active Rac1 or inhibiting Rho signalling. Rac1 has previously been reported to antagonize Rho activity directly 35 and regulate β1 integrin‐dependent motility in PDAC and colon cancer cells 46, 47. A recent study using computational modelling also identified a MEK‐driven feedback loop where inhibition of Eps8/Abi1/Sos1‐mediatied Rac1 activation following Eps8 knockdown leads to increased Rho activation 48. Unexpectedly, PDAC cells remained motile following inhibition of both Rac1 and Rho; several studies have similarly found that cell migration is possible in the absence of both GTPases 49, 50, 51.

Notably, Eps8 acted as a negative regulator of αvβ6‐dependent TGF‐β1 activation through the same mechanism (Figure 3), and both Rac1 and Sos1 knockdown similarly induced TGF‐β1 activation (Figure 5; supplementary material, Figure S8). The role of Rho kinases in αvβ6‐dependent TGF‐β activation has been implicated in several studies using ROCK inhibitors 11, 33, 34. It has also been hypothesized that increased cell tension generated by Rho activation results in a conformational change in LAP and exposure of active TGF‐β1 13. Our findings support this precept, and to the best of our knowledge, this is the first study to provide a direct link between Rac1/Rho activity and αvβ6‐dependent TGF‐β activation, showing that cells can apply mechanical forces to LAP and that the balance between Rac1 and Rho GTPase activation favours different αvβ6‐dependent cell functions.

Our results from 3D organotypic cultures highlight the problems of studying tumour cell motility in isolation and stress the important role of stromal cells in the invasive process. In Transwell® assays, Eps8 knockdown consistently suppressed PDAC motility; however, Eps8 knockdown in organotypic culture promoted the invasion of Panc0403 cells. This was modulated through increasing TGF‐β1 activation, which induced myofibroblastic transdifferentiation of fibroblasts/stellate cells (Figure 6; supplementary material, Figure S13). Rac1 or Sos1 knockdown produced a similar effect (supplementary material, Figure S14). This demonstrates how TGF‐β1 activation can indirectly promote invasion through stromal regulation. Given the notion that αvβ6‐targeting should be avoided in tumours that retain canonical signalling, it is noteworthy that αvβ6 inhibition significantly suppressed the invasion of Panc0403 cells that retain wild‐type SMAD4.

In summary, αvβ6 is a promising target for tumour therapy, but given its different functional effects, detailed knowledge of the underlying molecular mechanisms regulating these processes is required. We have shown that the balance in activation of Rho and Rac1 biases function towards TGF‐β1 activation or motility, respectively, with the presence or absence of Eps8 acting as a molecular switch that alters the balance of GTPase activation within the cell. In late‐stage disease, both αvβ6‐dependent functions are likely to be tumour‐promoting; however, we can speculate that in premalignant disease, the bias towards an Eps8‐expressing, invasive phenotype may help to promote malignant transformation.

## Author contributions statement

JT, VJ, CJH, MM, and SJF conceived experiments and analysed data. MR, DS, TB, MAL, PK, KAW, GS, and JFM provided technical and material support. JT, VJ, and GJT conceived and designed the study. DF and CJ contributed to the collection of clinical material and conception and design of the study. VJ and GJT jointly supervised the study and are co‐senior authors. All authors were involved in writing the paper and had final approval of the submitted and published versions.

**SUPPLEMENTARY MATERIAL ONLINE**

**Supplementary materials and methods**

**Supplementary figure legends**

**Figure S1.** Optimization of siRNA sequences

**Figure S2.** αvβ6 inhibition does not affect random PDAC cell migration towards BSA

**Figure S3.** TGF‐β1 activation by αvβ6‐positive PDAC cells

**Figure S4.** PDAC cell migration following Eps8 knockdown using additional siRNA sequences

**Figure S5.** Eps8 overexpression increases cell motility while it inhibits TGF‐β activation

**Figure S6.** Cell surface levels of β6 integrin and cell adhesion following Eps8 knockdown

**Figure S7.** Rac1 activation and PDAC cell migration following EGF stimulation

**Figure S8.** Sos1 expression in PDAC tissues and cells, and PDAC cell migration, invasion, and TGF‐β activation following Sos1 knockdown

**Figure S9.** Eps8 and Rac1 regulate cell motility and TGF‐β activation in the same pathway

**Figure S10.** Stress‐fibre formation in cells, where Eps8 siRNA was not effective, is limited

**Figure S11.** The cell‐permeable Rho inhibitor CT04 inhibits stress‐fibre formation in Eps8 knockdown cells

**Figure S12.** Rho inhibition with the specific inhibitor CT04 induces cell motility and inhibits TGF‐β activation

**Figure S13.** Organotypic invasion of BxPC3 and Panc0403 cells in the presence of primary pancreatic stellate cells

**Figure S14.** Organotypic invasion of Panc0403 cells following Rac1 and Sos1 knockdown

**Table S1.** Antibodies used in the study

**Table S2.** siRNAs used in the study

## Supporting information


**Supplementary materials and methods**

**Supplementary figure legends**

**Figure S1.** **Optimization of siRNA sequences.** BxPC3 cells were transfected with 30, 50 or 100 nm Eps8 (A, B), Rac1 (C), and Sos1 (D) siRNA; cells were harvested 24, 48 or 72 h post‐transfection; and protein (A, C, D) or mRNA (B) levels were tested using western blotting (A, C, D) or reverse transcription–quantitative PCR and expressed as fold change relative to Ctl. GAPDH was used as a reference transcript. (B) Results confirmed that the siRNA sequences produce significant down‐regulation of the proteins at each time point relevant to our functional assays. Equal loading on western blots was confirmed by HSC70. Numbers below the blots indicate the densitometry values measured using ImageJ normalized to HSC70 and expressed as a ratio of Ctl.

**Figure S2.** **αvβ6 inhibition does not affect PDAC cell migration towards BSA.** (A) Western blot showing αv expression in PDAC cell lines. Equal loading was confirmed by HSC70. (B) 50 000 PDAC cells were pretreated with 10 μg/ml of the αvβ6 blocking antibody 63G9 for 30 min before plating them into the top well of bovine serum albumin‐coated Transwell® migration inserts. The number of cells that migrated to the bottom wells was counted after an overnight incubation and no change in the number of migrating cells was detected upon pretreatment with the blocking antibody. Note the low number of migrating cells. Diagram represents the mean number of migrating cells per well ± SD; n = 3.

**Figure S3.** **αvβ6‐positive PDAC cells activate TGF‐β1.** PDAC cells (120 000) were plated on top of MLEC cells and TGF‐β1 activation was measured after an overnight incubation. The αvβ6‐positive Capan1, BxPC3, and Panc0403 cancer cells induced significant activation of TGF‐β1, while αvβ6‐negative SW1990 and SU86.86 cells did not. Diagram represents relative light units expressed as a % of Ctl ± SD; n = 6; *p < 0.05; ***p < 0.001; ****p < 0.001; ns = non‐significant.

**Figure S4.** **Eps8 knockdown using four siRNA sequences inhibits PDAC cell migration and induces TGF‐β1 activation.** (A) Transwell® migration of BxPC3 cells towards LAP was significantly inhibited by transfection with the Eps8 siRNA sequence used throughout the study (Eps8) and three alternative siRNA sequences targeting Eps8 (♯1‐2‐3). Diagram represents the mean number of migrating cells per well expressed as a % of Ctl (BSA) ± SD; n = 3; *p < 0.05. (B) Eps8 knockdown using four individual siRNA sequences induces activation of TGF‐β1 in BxPC3 cells measured by an MLEC TGF‐β activation assay. Diagram represents the mean relative light units expressed as a % of Ctl ± SD; n = 6; **p < 0.01; ***p < 0.001; ****p < 0.0001. Western blots confirm down‐regulation of Eps8 using RNA interference. Equal loading was confirmed by HSC70. Numbers below the blots indicate the densitometry values measured using ImageJ normalized to HSC70 and expressed as a ratio to Ctl.

**Figure S5.** **Eps8 overexpression increases cell motility while it inhibits TGF‐β activation.** (A) Capan1, BxPC3, and Panc0403 cells were transfected with empty vector (EV) or Eps8–EGFP 24 h before plating them into a Transwell® migration assay. Eps8 overexpression in all three cell lines significantly increased cell migration towards the αvβ6 ligand, LAP. Diagram represents the mean number of migrating cells per well expressed as a % of Ctl (BSA) ± SD (Capan1/Panc0403 plotted on left, BxPC3 plotted on right Y‐axis); n = 3; *p < 0.05; ***p < 0.001. (B) Capan1, BxPC3, and Panc0403 cells were transfected with empty vector (EV) or Eps8‐EGFP 24 h before plating them on top of MLEC cells. Eps8 overexpression significantly inhibited TGF‐β activation in all three cell lines. Diagram represents the mean relative light units expressed as a % of MLECs ± SD (Capan1/Panc0403 plotted on left, BxPC3 plotted on right Y‐axis); n = 6; **p < 0.01; ****p < 0.0001. (C) Eps8–EGFP expression was confirmed by western blotting. HSC70 was used as a loading control.

**Figure S6.** **Eps8 does not affect the cell surface levels of β6 integrin.** Cells were transfected with non‐targeting (Ctl) or Eps8‐targeting siRNA, and the cell surface levels of total (A) or active (B) β6 integrin were measured by FACS analysis 48 h post‐transfection using either anti‐β6 (620 W) (A) or anti‐active β6 (6.2G2) antibodies (B). Diagrams represent the mean fluorescence intensity expressed as a % of Ctl ± SD; n = 3; ns = non‐significant. (C) Cells were transfected with either non‐targeting (Ctl) or Eps8‐targeting siRNA, and cell adhesion on LAP was measured 48 h post‐transfection. Eps8 down‐regulation had no effect on αvβ6‐specific adhesion of PDAC cells. Diagrams represent the absorbance at 540 nm expressed as a % of Ctl (BSA) ± SD; n = 4; ns = non‐significant. Western blots confirmed down‐regulation of Eps8 following siRNA transfection. Equal loading was confirmed by HSC70. Numbers below the blots indicate the densitometry values measured using ImageJ normalized to HSC70 and expressed as a ratio to Ctl.

**Figure S7.** **EGF stimulation potentiates αvβ6 signalling and function.** (A) Western blot showing expression of EGFR in the αvβ6‐positive Capan1, BxPC3, and Panc0403 cancer cells. The SCC25 oral squamous cell carcinoma cell line was used as a positive control. Equal loading was confirmed by HSC70. (B) Stimulation of Capan1, BxPC3, and Panc0403 cells with 20 ng/ml EGF induced a significant increase in migration levels towards the αvβ6 integrin ligand LAP. This EGF‐induced migration was completely inhibited by the αvβ6 blocking antibody 63G9, confirming that EGF‐induced migration of PDAC cells was αvβ6‐dependent. Diagram represents the mean number of migrating cells per well expressed as a % of BSA ± SD; n = 3; *p < 0.05; **p < 0.01; ***p < 0.001; ****p < 0.0001. (C) Stimulation of Capan1, BxPC3, and Panc0403 cells with 20 ng/ml EGF induced a significant activation of the small GTPase Rac1, as evidenced by a GST‐PAK1‐CRIB pull‐down assay. Knockdown of αvβ6 completely blocked EGF‐induced Rac1 activation in all cell lines, confirming αvβ6 dependency. β6 knockdown in the same lysates was confirmed on separate western blots. Equal loading was confirmed by HSC70. Numbers below the blots indicate the densitometry values measured using ImageJ normalized to HSC70 and expressed as a ratio to Ctl.

**Figure S8.** **Sos1 is overexpressed in PDAC and promotes motility but inhibits TGF‐β activation.** (A) Representative image of the immunohistochemical staining of Sos1 in PDAC (ii; tumour) and surrounding (i; uninvolved) tissue. The table on the right shows the staining intensity using the QuickScore method. (B) Western blot showing Sos1 expression in three αvβ6‐positive PDAC cell lines. The human oral squamous cell carcinoma cell line VB6 was used as a positive control. Equal loading was confirmed by HSC70. (C) Results of a GST pull‐down assay using GST‐PAK1‐CRIB‐coated Sepharose beads showing that Sos1 knockdown completely inhibits EGF‐induced Rac1 activation compared with cells transfected with non‐targeting (Ctl) siRNA in Capan1, BxPC3, and Panc0403 cells. The western blot for Capan1 cells originated from the same experiment presented in Figure 3A. Eps8 and Sos1 knockdown in the same lysates was confirmed on separate western blots. Equal loading was confirmed by HSC70. Numbers below the blots indicate the densitometry values measured using ImageJ normalized to HSC70 and are expressed as a ratio to Ctl. (D) Sos1 down‐regulation by RNA interference significantly inhibits Transwell® migration of Capan1, BxPC3, and Panc0403 cells towards the αvβ6 integrin ligand LAP compared with non‐targeting (Ctl) siRNA. Diagram represents the mean number of migrated cells per well expressed as a % of Ctl (LAP) ± SD; n = 3; *p < 0.05; **p < 0.01. (E) Invasion of Capan1, BxPC3, and Panc0403 cells through Matrigel‐coated Transwells® was significantly inhibited by down‐regulation of Sos1 using RNA interference. Diagram represents the mean number of invaded cells per well expressed as a % of Ctl ± SD; n = 4; *p < 0.05; **p < 0.01; ***p < 0.001. (F) Sos1 knockdown induced activation of TGF‐β1 compared with non‐targeting (Ctl) siRNA‐transfected cells in an MLEC TGF‐β activation assay. Sos1‐induced TGF‐β activation was inhibited by the αvβ6 blocking antibody 63G9. Diagram represents the mean relative light units expressed as a % of MLECs ± SD (Capan1/BxPC3 plotted on left, Panc0403 plotted on right Y‐axis); n = 6; **p < 0.01; ***p < 0.001; ****p < 0.0001. Western blots in D–F confirmed down‐regulation of Sos1 using RNA interference. Equal loading was confirmed by HSC70. Numbers below the blots indicate the densitometry values measured using ImageJ normalized to HSC70 and expressed as a ratio to Ctl.

**Figure S9.** **Eps8 and Rac1 regulate cell motility and TGF‐β activation in the same pathway.** (A) Transwell® migration of Capan1, BxPC3, and Panc0403 cells towards LAP is inhibited by overnight pretreatment with 50 μm of the Rac1 inhibitor, NSC23766 (Raci). Diagram represents the mean number of migrated cells per well expressed as a % of Ctl (BSA) ± SD; n = 3; **p < 0.01; ***p < 0.001. (B) Transwell® migration of Capan1 cells towards LAP was inhibited to the same extent by down‐regulation of Eps8 or Rac1 and simultaneous down‐regulation of both proteins. Diagram represents the mean number of migrated cells per well expressed as a % of Ctl (BSA) ± SD; n = 3; **p < 0.01; ***p < 0.001. Western blots confirm down‐regulation of Eps8 and Rac1. Equal loading was confirmed by HSC70. Numbers below the blots indicate the densitometry values for Eps8 (D˙Eps8) and Rac1 (D.Rac1) knockdown measured using ImageJ normalized to HSC70 and expressed as a ratio to Ctl. (C) Capan1, BxPC3, and Panc0403 cells were pretreated overnight by 50 μm of the Rac1 inhibitor NSC23766 (Raci), after which they were plated on top of MLEC cells in the absence of the inhibitor. Rac1 inhibition significantly increased TGF‐β activation in all three cell lines. Diagram represents the mean relative light units expressed as a % of MLECs ± SD; n = 6; **p < 0.01; ***p < 0.001; ****p < 0.0001. (D) Eps8, Rac1 or Eps8 and Rac1 siRNA induced significantly increased TGF‐β activation in Capan1 cells. Diagram represents the mean relative light units expressed as a % of MLECs ± SD; n = 6; **p < 0.01; ***p < 0.001. Western blots in B confirmed down‐regulation of Eps8 and Rac1 using RNA interference.

**Figure S10.** **Stress‐fibre formation was reduced in cells with incomplete knockdown of Eps8 and Rac1.** Capan1 cells were transfected with Eps8 or Rac1 siRNA, plated on 0.5 μg/ml LAP‐coated coverslips, and after an overnight incubation stained with phalloidin‐FITC (red) to visualize stress‐fibre formation or anti‐Eps8 or Rac1 antibodies (green) to detect the level of knockdown. DAPI (blue) was used as a nuclear counterstain. Cells with absent or very low levels of Eps8 (A, bottom panels) and Rac1 (B, bottom panels) expression showed increased stress‐fibre formation, whereas cells in which Eps8 (A, top panels) and Rac1 (B, top panels) down‐regulation was incomplete did not produce stress fibres. Images were captured at the same microscope setting and exposure was uniformly enhanced across images to aid better visibility. Representative images are shown. Western blots confirm down‐regulation of Eps8 or Rac1 following siRNA transfection. Equal loading was confirmed by HSC70. Numbers below the blots indicate the densitometry values measured using ImageJ normalized to HSC70 and expressed as a ratio to Ctl.

**Figure S11.** **The cell‐permeable Rho inhibitor CT04 inhibits stress‐fibre formation in Eps8 knockdown cells.** Capan1 cells transfected with Eps8 siRNA were plated on 0.5 μg/ml LAP‐coated coverslips in the presence of 1% serum until they had fully adhered and spread. Medium on the cells was changed to serum‐free medium and cells were incubated in the absence (top panel) or presence (bottom panel) of 0.5 μg/ml CT04 Rho inhibitor. After overnight incubation, cells were fixed and stained with phalloidin‐FITC (red) to visualize stress‐fibre formation. DAPI (blue) was used as a nuclear counterstain. Exposure was uniformly enhanced across images to aid better visibility. A representative image is shown.

**Figure S12.** **Rho inhibition induces cell motility and inhibits TGF‐β activation.** (A) Capan1, BxPC3, and Panc0403 cells were pretreated with 0.5 μg/ml of the CT04 Rho inhibitor (Rhoi) before plating them into a Transwell® migration assay. Migration towards LAP of all three cell lines was significantly increased by Rho inhibition. Diagram represents the mean number of migrated cells per well expressed as a % of Ctl (BSA) ± SD (Capan1/Panc0403 plotted on left, BxPC3 plotted on right Y‐axis); n = 3; *p < 0.05; **p < 0.01. (B) Capan1, BxPC3, and Panc0403 cells were pretreated by 0.5 μg/ml of the CT04 Rho inhibitor (Rhoi), after which they were plated on top of MLEC cells in the absence of the inhibitor. Rho inhibition significantly inhibited TGF‐β activation in all three cell lines. Diagram represents the mean relative light units expressed as a % of MLECs ± SD (Capan1 plotted on left, BxPC3/Panc0403 plotted on right Y‐axis); n = 6; **p < 0.01; ****p < 0.0001.

**Figure S13.** **BxPC3 and Panc0403 cells invade differently in the presence of primary pancreatic stellate cells.** BxPC3 and Panc0403 cells were transfected with either non‐targeting (Ctl) or Eps8‐targeting siRNA, and organotypic invasion assays were performed in the presence of primary pancreatic stellate cells over a period of 12 days. While invasion of BxPC3 cells was significantly inhibited by down‐regulation of Eps8 compared with Ctl cells, invasion of Panc0403 cells was significantly increased. A representative image of cytokeratin‐stained sections is shown. Diagram shows the mean invasion depth of three independent sections analysed by ImageJ software expressed as a % of Ctl ± SD; n = 3; *p < 0.05; **p < 0.01.

**Figure S14.** **Rac1 and Sos1 inhibit the invasion of Panc0403 but not that of BxPC3 cells.** (Ai) BxPC3 and Panc0403 cells were transfected with either non‐targeting (Ctl) or Rac1‐targeting siRNA, and organotypic invasion assays were performed in the presence of HFFF2 fibroblasts over a period of 12 days. While down‐regulation of Rac1 inhibited the invasion of BxPC3 cells compared with Ctl cells, it induced a significant level of invasion in Panc0403 cells. A representative image of a cytokeratin‐stained section is shown. (Aii) Diagrams show the mean invasion depth of three independent sections analysed by ImageJ software expressed as % of Ctl ± SD; n = 3; **p < 0.01; ***p < 0.001. Western blots confirmed down‐regulation of Rac1. Equal loading was confirmed by HSC70. (Bi) BxPC3 and Panc0403 cells were transfected with either non‐targeting (Ctl) or Sos1‐targeting siRNA, and organotypic invasion assays were performed in the presence of HFFF2 fibroblasts over a period of 12 days. While down‐regulation of Sos1 inhibited the invasion of BxPC3 cells compared with Ctl cells, it induced a significant level of invasion in Panc0403 cells. A representative image of a cytokeratin‐stained section is shown. (Bii) Diagrams show the mean invasion depth of three independent sections analysed by ImageJ software expressed as a % of Ctl ± SD; n = 3; *p < 0.05; **p < 0.01. Western blots confirmed down‐regulation of Sos1. Equal loading was confirmed by HSC70. Numbers below the blots in Aii and Bii indicate the densitometry values measured using ImageJ normalized to HSC70 and expressed as a ratio to Ctl.

**Table S1.** Antibodies used in the study

**Table S2.** siRNAs used in the study
