# Supplementary material for: Pro‐migratory and TGF‐β‐activating functions of αvβ6 integrin in pancreatic cancer are differentially regulated via an Eps8‐dependent GTPase switch
Source: J Pathol. 2017 Aug 7;243(1):37–50. doi: 10.1002/path.4923 (PMC5601247; doi:10.1002/path.4923)
Supplement: Supplementary file 1 — Supplementary materials and methods [file PATH-243-37-s018.docx]

**Supplementary materials and methods**

**Reverse transcription and real-time PCR**

Cells were transfected with Eps8 siRNA using Oligofectamine™ transfection reagent and 24, 48 or 72 h post-transfection cells were harvested and either lysed in NP40 lysis buffer to extract protein or processed using the RNEasy mini kit (Qiagen, Hilden, Germany) to isolate total RNA. An aliquot of 500 ng of total RNA was reverse-transcribed to synthesize cDNA using the High Capacity cDNA Reverse Transcription Kit (Applied Biosystems, Foster City, CA, USA), according to the manufacturer’s instructions. Real-time PCR using Power SyBr^®^ Green (Life Technologies, Carlsbad, CA, USA) was performed with the 7500 Real-time PCR system (Applied Biosystems). The expression of the *EPS8* gene (forward primer, CGACCAAGGGACTTTGAGA; reverse primer, GCACATCTCTGTCAATGCGG) relative to the reference gene, *GAPDH* (forward primer, AGCAATGCCTGCACCACCAAC; reverse primer, CCGGAGGGGCCATCCACAGTCT), was calculated using the *∆∆*Ct method.

**Transfection with Eps8–EGFP**

Cells were plated at a density of 300 000 cells per well in six-well plates overnight and transfected with either 3 μg of empty vector control (pEGFP-C2) or EGFP-tagged mouse Eps8 using Fugene HD transfection reagent (Promega) and 24 h post-transfection cells were used for either Transwell^®^ migration or MLEC TGF-β activation assays.

**FACS analysis**

Cell surface expression of the αvβ6 integrin was quantified by flow cytometry. Total and active cell surface levels of integrins were detected using 620w and 6.2G2 antibodies (Biogen Idec Inc, Cambridge, MA, USA) for 1 h at 4°C, followed by incubation with Alexa-488 conjugated secondary antibodies (Invitrogen) for 45 min at 4°C in the dark. Labelled cells were scanned on a FACS Canto II cytometer (BD Biosciences, San Diego, CA, USA) by acquiring 1 × 10^4^ events. Analysis was performed using Cellquest Pro software.

**Adhesion assay**

Cells were transfected with non-targeted or Eps8-targeting siRNA for 48 h and then pretreated or not with the αvβ6 blocking antibody 63G9 (Biogen Idec) for 30 min at 4°C and plated on TGF-β1 LAP-coated 96-well plates in quadruplicates. After 1 h incubation at 37°C, cells were fixed in 1% glutaraldehyde (Sigma-Aldrich, Gillingham, Dorset, UK) and stained with crystal violet. Stained cells were washed with PBS and the remaining crystal violet was dissolved in 50% acetic acid. Absorbance was determined using a Varioskan plate reader at 540 nm.
