# Supplementary material for: Pro‐migratory and TGF‐β‐activating functions of αvβ6 integrin in pancreatic cancer are differentially regulated via an Eps8‐dependent GTPase switch
Source: J Pathol. 2017 Aug 7;243(1):37–50. doi: 10.1002/path.4923 (PMC5601247; doi:10.1002/path.4923)
Supplement: Supplementary file 2 — Supplementary figure legends [file PATH-243-37-s001.docx]

**Supplementary Figure Legends**

**Figure S1. Optimisation of siRNA sequences.** BxPC3 cells were transfected with 30, 50 or 100 nM Eps8 (A-B), Rac1 (C) and Sos1 (D) siRNA and cells were harvested 24, 48 or 72 h post-transfection and protein (A, C, D) or mRNA (B) levels were tested using western blotting (A, C, D) or Reverse Transcription - quantitative PCR and expressed as fold change relative to Ctl. *GAPDH* was used as a reference transcript. (B). Results confirmed that the siRNA sequences produce significant down-regulation of the proteins at each time point relevant to our functional assays. Equal loading on western blots was confirmed by HSC70. Numbers below blots indicate the densitometry values measured using ImageJ normalised to HSC70 and expressed as a ratio of Ctl.

**Figure S2. αvβ6 inhibition does not affect PDAC cell migration towards BSA.** (A) Western blot showing αv expression in PDAC cell lines. Equal loading was confirmed by HSC70. (B) 50 000 PDAC cells were pre-treated with 10 μg/ml of the αvβ6 blocking antibody 63G9 for 30 min before plating them into the top well of bovine serum albumin-coated Transwell^®^ migration inserts. The number of cells migrated to the bottom wells was counted after an overnight incubation and no change in the number of migrating cells was detected upon pre-treatment with the blocking antibody. Note the low number of migrating cells. Diagram represents mean number of migrating cells/well +/- SD; n=3.

**Figure S3. αvβ6-positive PDAC cells activate TGF-β1.** PDAC cells (120 000) were plated on top of MLEC cells and TGF-β1 activation was measured after an overnight incubation. The αvβ6-positive Capan1, BxPC3 and Panc0403 cancer cells induced significant activation of TGF-β1, while αvβ6-negative SW1990 and SU86.86 cells did not. Diagram represents Relative Light Units expressed as % of Ctl +/- SD, n=6, * *p*<0.05; *** *p*<0.001; **** *p*<0.001; ns = non-significant.

**Figure S4. Eps8 knockdown using four siRNA sequences inhibits PDAC cell migration and induces TGF-β1 activation.** (A) Transwell^®^ migration of BxPC3 cells towards LAP was significantly inhibited by transfection with the Eps8 siRNA sequence used throughout the study (Eps8) and three alternative siRNA sequences targeting Eps8 (♯1-2-3). Diagram represents mean number of migrating cells/well expressed as % of Ctl (BSA) +/- SD, n=3, * *p*<0.05. (B) Eps8 knockdown using four individual siRNA sequences induces activation of TGF-β1 in BxPC3 cells measured by an MLEC TGF-β activation assay. Diagram represents mean Relative Light Units expressed as % of Ctl +/- SD, n=6, ** *p*<0.01; ****p*<0.001; *****p*<0.0001. Western blots confirm down-regulation of Eps8 using RNA interference. Equal loading was confirmed by HSC70. Numbers below blots indicate the densitometry values measured using ImageJ normalised to HSC70 and expressed as a ratio to Ctl.

**Figure S5. Eps8 overexpression increases cell motility while inhibits TGF-β activation.** (A) Capan1, BxPC3 and Panc0403 cells were transfected with empty vector (EV) or Eps8-EGFP 24 h before plating them into a Transwell^®^ migration assay. Eps8 overexpression in all three cell lines significantly increased cell migration towards the αvβ6 ligand, LAP. Diagram represents mean number of migrating cells/well expressed as % of Ctl (BSA) +/- SD (Capan1/Panc0403 plotted on left, BxPC3 plotted on right Y-axis), n=3, * *p*<0.05; ****p*<0.001. (B) Capan1, BxPC3 and Panc0403 cells were transfected with empty vector (EV) or Eps8-EGFP 24 h before plating them on top of MLEC cells. Eps8 overexpression significantly inhibited TGF-β activation in all three cell lines. Diagram represents mean Relative Light Units expressed as % of MLEC +/- SD (Capan1/Panc0403 plotted on left, BxPC3 plotted on right Y-axis), n=6, ** *p*<0.01; *****p*<0.0001. (C) Eps8-EGFP expression was confirmed by western blotting. HSC70 was used as loading control.

**Figure S6. Eps8 does not affect cell surface levels of β6 integrin.** Cells were transfected with non-targeting (Ctl) or Eps8-targeting siRNA and cell surface levels of total (A) or active (B) β6 integrin were measured by FACS analysis 48 h post-transfection using either anti-β6 (620W) (A) or anti-active β6 (6.2G2) antibodies (B). Diagrams represent Mean Fluorescence Intensity expressed as % of Ctl +/- SD, n=3, ns = non-significant. (C) Cells were transfected with either non-targeting (Ctl) or Eps8-targeting siRNA and cell adhesion on LAP was measured 48 h post-transfection. Eps8 down-regulation had no effect on αvβ6-specific adhesion of PDAC cells. Diagrams represent absorbance at 540 nm expressed as % of Ctl (BSA) +/- SD, n=4, ns = non-significant. Western blots confirmed down-regulation of Eps8 following siRNA transfection. Equal loading was confirmed by HSC70. Numbers below blots indicate the densitometry values measured using ImageJ normalised to HSC70 and expressed as a ratio to Ctl.

**Figure S7. EGF stimulation potentiates αvβ6 signalling and function.** (A) Western blot showing expression of EGFR in the αvβ6-positive Capan1, BxPC3 and Panc0403 cancer cells. The SCC25 oral squamous cell carcinoma cell line was used as positive control. Equal loading was confirmed by HSC70. (B) Stimulation of Capan1, BxPC3 and Panc0403 cells with 20 ng/ml EGF induced a significant increase in migration levels towards the αvβ6 integrin ligand LAP. This EGF-induced migration was completely inhibited by the αvβ6 blocking antibody 63G9 confirming that EGF-induced migration of PDAC cells was αvβ6-dependent. Diagram represents mean number of migrating cells/well expressed as % of BSA +/- SD, n=3, * *p*<0.05; ** *p*<0.01; ****p*<0.001 *****p*<0.0001. (C) Stimulation of Capan1, BxPC3 and Panc0403 cells with 20 ng/ml EGF induced a significant activation of the small GTPase Rac1 as evidenced by a GST-PAK1-CRIB pull-down assay. Knockdown of αvβ6 completely blocked EGF-induced Rac1 activation in all cell lines confirming αvβ6 dependency. β6 knockdown in the same lysates was confirmed on separate western blots. Equal loading was confirmed by HSC70. Numbers below blots indicate the densitometry values measured using ImageJ normalised to HSC70 and expressed as a ratio to Ctl.

**Figure S8. Sos1 is overexpressed in PDAC, promotes motility but inhibits TGF-β activation.** (A) A representative image of immunohistochemical staining of Sos1 in PDAC (ii; tumour) and surrounding (i; uninvolved) tissue. Table showing the staining intensity using the QuickScore method. (B) Western blot showing Sos1 expression in three αvβ6-positive PDAC cell lines. The human oral squamous cell carcinoma cell line, VB6 was used as positive control. Equal loading was confirmed by HSC70. (C) Results of a GST-pull-down assay using GST-PAK1-CRIB-coated Sepharose beads showing that Sos1 knockdown completely inhibits EGF-induced Rac1 activation compared to cells transfected with non-targeting (Ctl) siRNA in Capan1, BxPC3 and Panc0403 cells. Western blot for Capan1 cells originated from the same experiment presented in Figure 3A. Eps8 and Sos1 knockdown in the same lysates was confirmed on separate western blots. Equal loading was confirmed by HSC70. Numbers below blots indicate the densitometry values measured using ImageJ normalised to HSC70 and expressed as a ratio to Ctl. (D) Sos1 down-regulation by RNA interference significantly inhibits Transwell^®^ migration of Capan1, BxPC3 and Panc0403 cells towards the αvβ6 integrin ligand, LAP compared to non-targeting (Ctl) siRNA. Diagram represents mean number of migrated cells/well expressed as % of Ctl (LAP) +/- SD, n=3, * *p*<0.05; ** *p*<0.01. (E) Invasion of Capan1, BxPC3 and Panc0403 cells through Matrigel-coated Transwells^®^ was significantly inhibited by down-regulation of Sos1 using RNA interference. Diagram represents mean number of invaded cells/well expressed as a % of Ctl +/- SD, n=4, * *p*<0.05; ** *p*<0.01; *** *p*<0.001. (F) Sos1 knockdown induced activation of TGF-β1 compared to non-targeting (Ctl) siRNA-transfected cells in an MLEC TGF-β activation assay. Sos1 knockdown-induced TGF-β activation was inhibited by the αvβ6 blocking antibody, 63G9. Diagram represents mean Relative Light Units expressed as % of MLEC +/- SD, (Capan1/BxPC3 plotted on left, Panc0403 plotted on right Y-axis), n=6, ** *p*<0.01; ****p*<0.001; *****p*<0.0001. Western blots in D-F confirmed down-regulation of Sos1 using RNA interference. Equal loading was confirmed by HSC70. Numbers below blots indicate the densitometry values measured using ImageJ normalised to HSC70 and expressed as a ratio to Ctl.

**Figure S9. Eps8 and Rac1 regulate cell motility and TGF-β activation in the same pathway.** (A) Transwell^®^ migration of Capan1, BxPC3 and Panc0403 cells towards LAP is inhibited by overnight pre-treatment with 50 μM of the Rac1 inhibitor, NSC23766 (Raci). Diagram represents mean number of migrated cells/well expressed as % of Ctl (BSA) +/- SD, n=3, ** *p*<0.01; ****p*<0.001. (B) Transwell^®^ migration of Capan1 cells towards LAP was inhibited to the same extent by down-regulation of Eps8 or Rac1 and simultaneous down-regulation of both proteins. Diagram represents mean number of migrated cells/well expressed as % of Ctl (BSA) +/- SD, n=3, ** *p*<0.01; ****p*<0.001. Western blots confirm down-regulation of Eps8 and Rac1. Equal loading was confirmed by HSC70. Numbers below blots indicate the densitometry values for Eps8 (D.Eps8) and Rac1 (D.Rac1) knockdown measured using ImageJ normalised to HSC70 and expressed as a ratio to Ctl. (C) Capan1, BxPC3 and Panc0403 cells were pre-treated overnight by 50 μM of Rac1 inhibitor, NSC23766 (Raci) after which they were plated on top of MLEC cells in the absence of the inhibitor. Rac1 inhibition significantly increased TGF-β activation in all three cell lines. Diagram represents mean Relative Light Units expressed as % of MLEC +/- SD, n=6, ** *p*<0.01; ****p*<0.001; **** *p*<0.0001. (D) Eps8, Rac1 or Eps8 and Rac1 siRNA induced significantly increased TGF-β activation in Capan1 cells. Diagram represents mean Relative Light Units expressed as a % of MLEC +/- SD, n=6, ** *p*<0.01; ****p*<0.001. Western blots in (B) confirmed down-regulation of Eps8 and Rac1 using RNA interference.

**Figure S10. Stress-fibre formation was reduced in cells with incomplete knockdown of Eps8 and Rac1.** Capan1 cells were transfected with Eps8 or Rac1 siRNA, plated on 0.5 μg/ml LAP-coated coverslips and after an overnight incubation stained with Phalloidin-FITC (red) to visualize stress-fibre formation or anti-Eps8 or Rac1 antibodies (green) to detect the level of knockdown. DAPI (blue) was used as nuclear counter-stain. Cells with absent or very low levels of Eps8 (A, bottom panels) and Rac1 (B, bottom panels) expression showed increased stress-fibre formation, whereas cells in which Eps8 (A, top panels) and Rac1 (B, top panels) down-regulation was incomplete did not produce stress-fibres. Images were captured at the same microscope setting and exposure was uniformly enhanced across images to aid better visibility. Representative images are shown. Western blots confirm down-regulation of Eps8 or Rac1 following siRNA transfection. Equal loading was confirmed by HSC70. Numbers below blots indicate the densitometry values measured using ImageJ normalised to HSC70 and expressed as a ratio to Ctl.

**Figure S11. The cell permeable Rho inhibitor CT04 inhibits stress-fibre formation in Eps8 knockdown cells.** Capan1 cells transfected with Eps8 siRNA were plated on 0.5 μg/ml LAP-coated coverslips in the presence of 1% serum until they have fully adhered and spread. Medium on the cells was changed to serum-free medium and cells were incubated in the absence (top panel) or presence (bottom panel) of 0.5 μg/ml CT04 Rho inhibitor. After overnight incubation cells were fixed and stained with Phalloidin-FITC (red) to visualize stress-fibre formation. DAPI (blue) was used as nuclear counterstain. Exposure was uniformly enhanced across images to aid better visibility. A representative image is shown.

**Figure S12. Rho inhibition induces cell motility and inhibits TGF-β activation.** (A) Capan1, BxPC3 and Panc0403 cells were pre-treated with 0.5 μg/ml of the CT04 Rho inhibitor (Rhoi) before plating them into a Transwell^®^ migration assay. Migration towards LAP of all three cell lines was significantly increased by Rho inhibition. Diagram represents mean number of migrated cells/well expressed as a % of Ctl (BSA) +/- SD (Capan1/Panc0403 plotted on left, BxPC3 plotted on right Y-axis), n=3, * *p*<0.05; ***p*<0.01. (B) Capan1, BxPC3 and Panc0403 cells were pre-treated by 0.5 μg/ml of the CT04 Rho inhibitor (Rhoi) after which they were plated on top of MLEC cells in the absence of the inhibitor. Rho inhibition significantly inhibited TGF-β activation in all three cell lines. Diagram represents mean Relative Light Units expressed as a % of MLEC +/- SD (Capan1 plotted on left, BxPC3/Panc0403 plotted on right Y-axis), n=6, ** *p*<0.01; *****p*<0.0001.

**Figure S13. BxPC3 and Panc0403 cells invade differently in the presence of primary pancreatic stellate cells.** BxPC3 and Panc0403 cells were transfected with either non-targeting (Ctl) or Eps8-targeting siRNA and organotypic invasion assays were performed in the presence of primary pancreatic stellate cells over a period of 12 days. While invasion of BxPC3 cells was significantly inhibited by down-regulation of Eps8 compared to Ctl cells, invasion of Panc0403 cells was significantly increased. A representative image of cytokeratin-stained sections is shown. Diagram shows mean invasion depth of three independent sections analysed by ImageJ software expressed as a % of Ctl +/- SD. n=3, * *p*<0.05; ** *p*<0.01.

**Figure S14. Rac1 and Sos1 inhibits invasion of Panc0403 but not of BxPC3 cells.** (Ai) BxPC3 and Panc0403 cells were transfected with either non-targeting (Ctl) or Rac1-targeting siRNA and organotypic invasion assays were performed in the presence of HFFF2 fibroblasts over a period of 12 days. While down-regulation of Rac1 inhibited invasion of BxPC3 cells compared to Ctl cells, it induced a significant level of invasion in Panc0403 cells. A representative image of cytokeratin-stained section is shown. (Aii) Diagrams show mean invasion depth of three independent sections analysed by ImageJ software expressed as % of Ctl +/- SD. n=3, ** *p*<0.01; *** *p*<0.001. Western blots confirmed down-regulation of Rac1. Equal loading was confirmed by HSC70. (Bi) BxPC3 and Panc0403 cells were transfected with either non-targeting (Ctl) or Sos1-targeting siRNA and organotypic invasion assays were performed in the presence of HFFF2 fibroblasts over a period of 12 days. While down-regulation of Sos1 inhibited invasion of BxPC3 cells compared to Ctl cells, it induced a significant level of invasion in Panc0403 cells. A representative image of cytokeratin-stained section is shown. (Bii) Diagrams show mean invasion depth of three independent sections analysed by ImageJ software expressed as a % of Ctl +/- SD. n=3, * *p*<0.05; ** *p*<0.01. Western blots confirmed down-regulation of Sos1. Equal loading was confirmed by HSC70. Numbers below blots in (Aii) and (Bii) indicate the densitometry values measured using ImageJ normalised to HSC70 and expressed as a ratio to Ctl.
