# Supplementary figures and images for: Pro‐migratory and TGF‐β‐activating functions of αvβ6 integrin in pancreatic cancer are differentially regulated via an Eps8‐dependent GTPase switch
Source: J Pathol. 2017 Aug 7;243(1):37–50. doi: 10.1002/path.4923 (PMC5601247; doi:10.1002/path.4923)

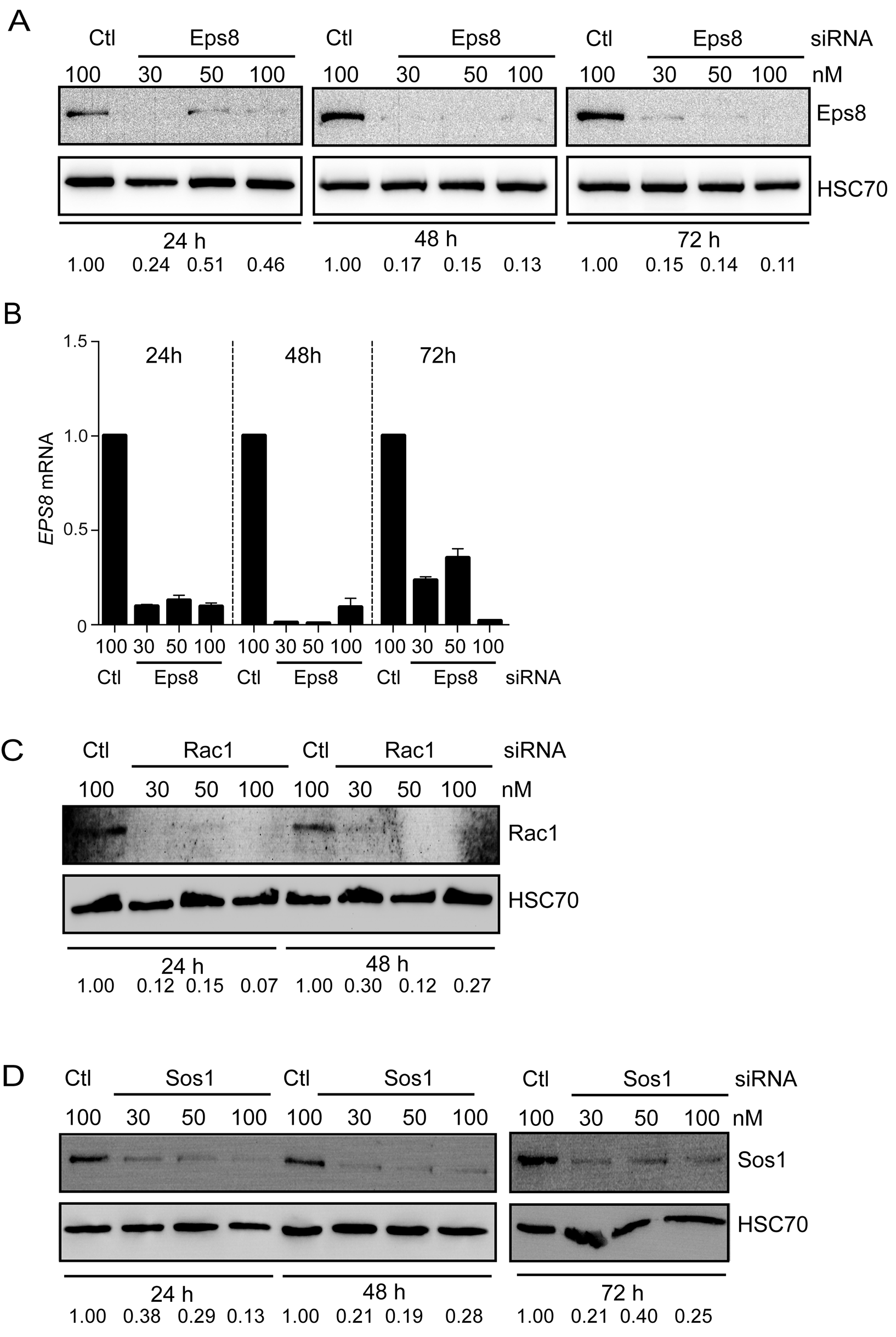

Supplement: Supplementary file 3 — Figure S1. Optimization of siRNA sequences. BxPC3 cells were transfected with 30, 50 or 100 nm Eps8 (A, B), Rac1 (C), and Sos1 (D) siRNA; cells were harvested 24, 48 or 72 h post‐transfection; and protein (A, C, D) or mRNA (B) levels were tested using western blotting (A, C, D) or reverse transcription–quantitative PCR and expressed as fold change relative to Ctl. GAPDH was used as a reference transcript. (B) Results confirmed that the siRNA sequences produce significant down‐regulation of the proteins at each time point relevant to our functional assays. Equal loading on western blots was confirmed by HSC70. Numbers below the blots indicate the densitometry values measured using ImageJ normalized to HSC70 and expressed as a ratio of Ctl. [file PATH-243-37-s003.tif]

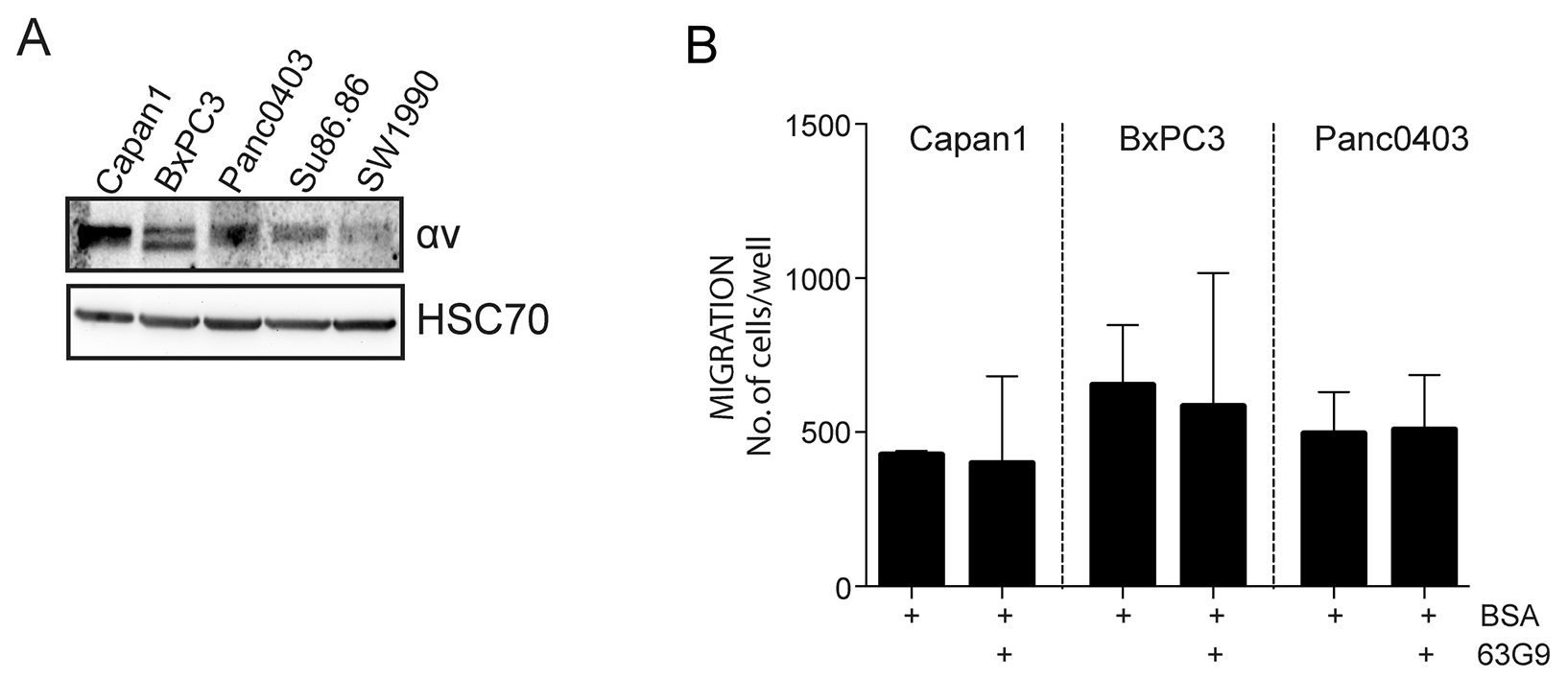

Supplement: Supplementary file 4 — Figure S2. αvβ6 inhibition does not affect PDAC cell migration towards BSA. (A) Western blot showing αv expression in PDAC cell lines. Equal loading was confirmed by HSC70. (B) 50 000 PDAC cells were pretreated with 10 μg/ml of the αvβ6 blocking antibody 63G9 for 30 min before plating them into the top well of bovine serum albumin‐coated Transwell® migration inserts. The number of cells that migrated to the bottom wells was counted after an overnight incubation and no change in the number of migrating cells was detected upon pretreatment with the blocking antibody. Note the low number of migrating cells. Diagram represents the mean number of migrating cells per well ± SD; n = 3. [file PATH-243-37-s010.tif]

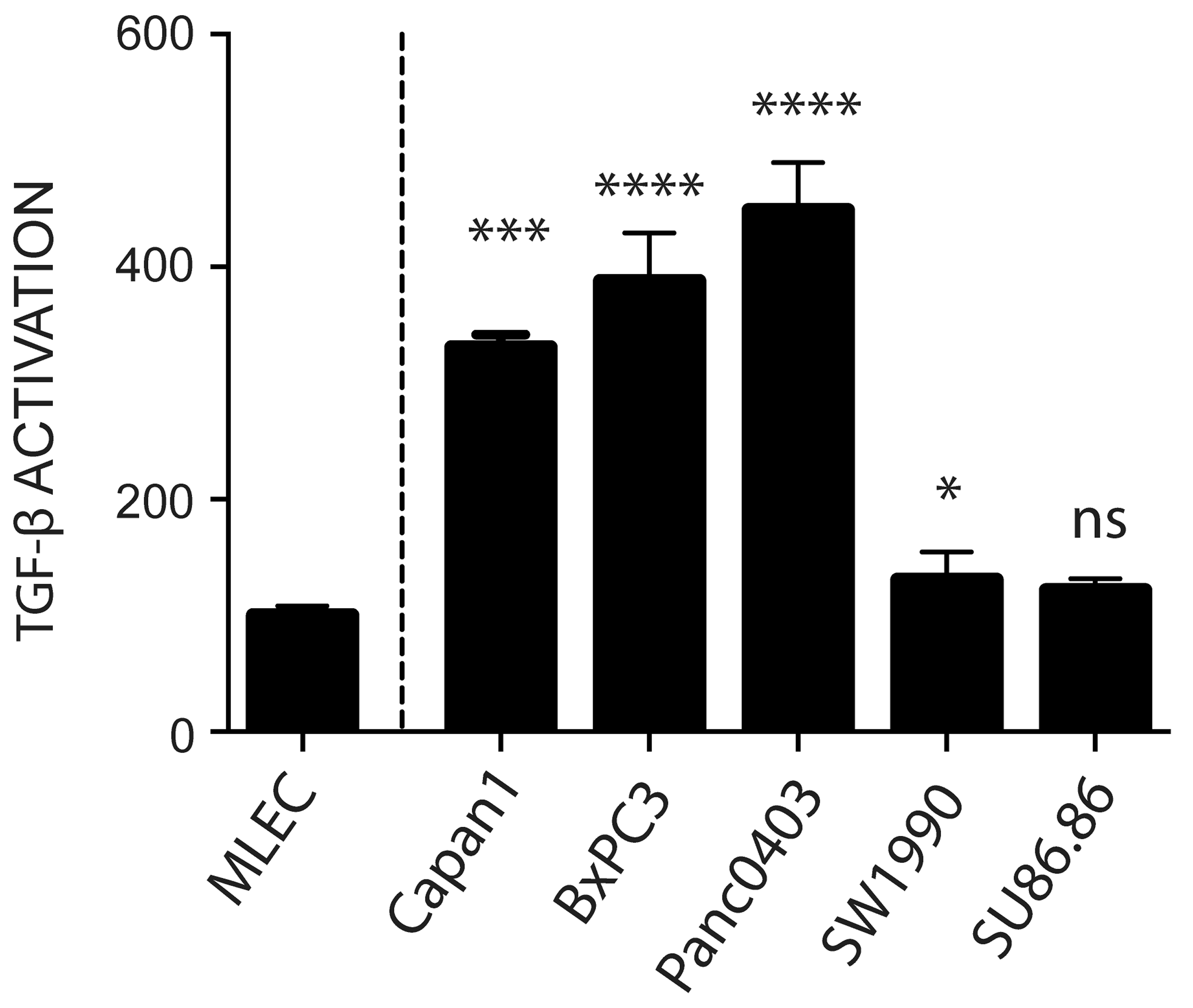

Supplement: Supplementary file 5 — Figure S3. αvβ6‐positive PDAC cells activate TGF‐β1. PDAC cells (120 000) were plated on top of MLEC cells and TGF‐β1 activation was measured after an overnight incubation. The αvβ6‐positive Capan1, BxPC3, and Panc0403 cancer cells induced significant activation of TGF‐β1, while αvβ6‐negative SW1990 and SU86.86 cells did not. Diagram represents relative light units expressed as a % of Ctl ± SD; n = 6; *p < 0.05; ***p < 0.001; ****p < 0.001; ns = non‐significant. [file PATH-243-37-s007.tif]

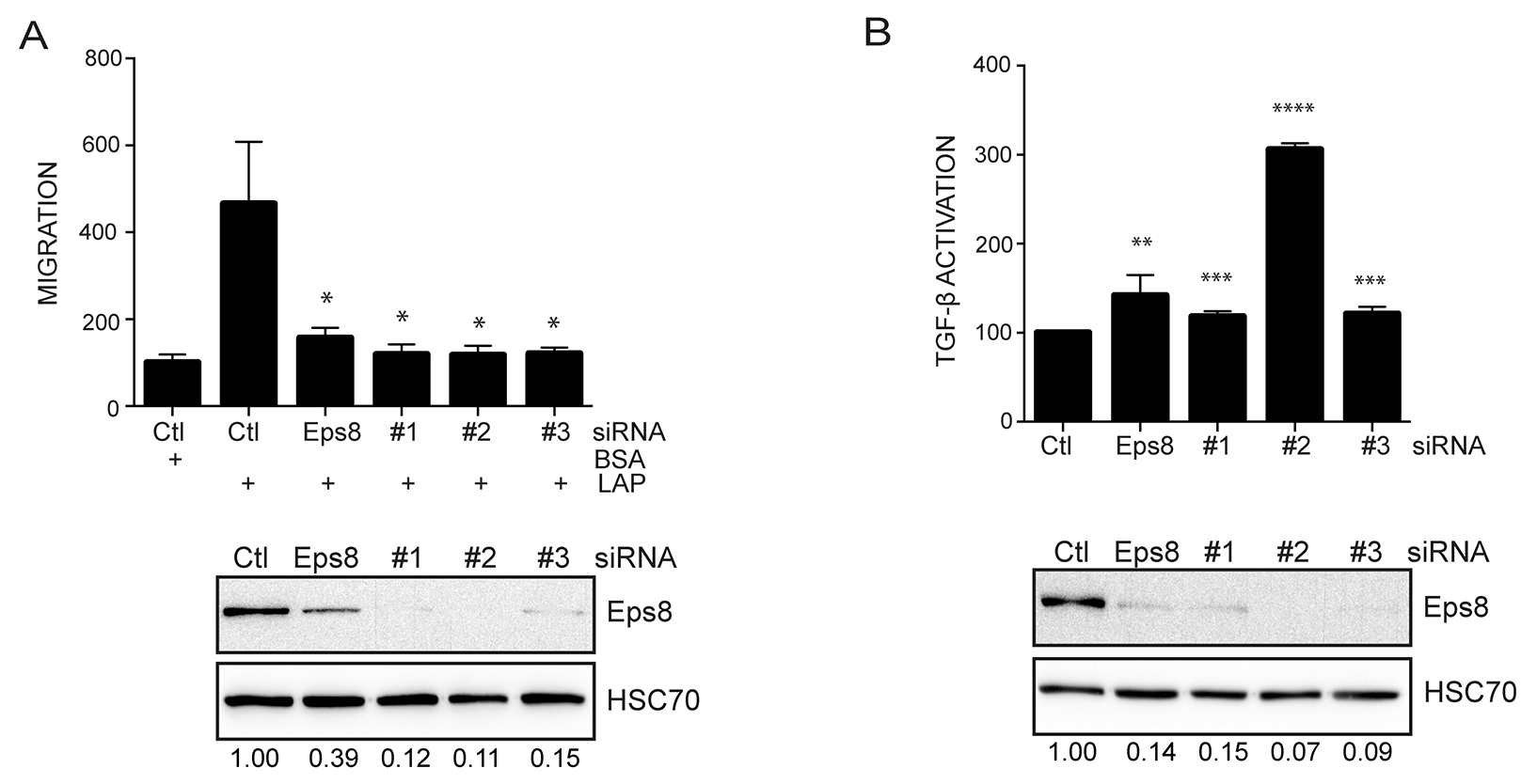

Supplement: Supplementary file 6 — Figure S4. Eps8 knockdown using four siRNA sequences inhibits PDAC cell migration and induces TGF‐β1 activation. (A) Transwell® migration of BxPC3 cells towards LAP was significantly inhibited by transfection with the Eps8 siRNA sequence used throughout the study (Eps8) and three alternative siRNA sequences targeting Eps8 (♯1‐2‐3). Diagram represents the mean number of migrating cells per well expressed as a % of Ctl (BSA) ± SD; n = 3; *p < 0.05. (B) Eps8 knockdown using four individual siRNA sequences induces activation of TGF‐β1 in BxPC3 cells measured by an MLEC TGF‐β activation assay. Diagram represents the mean relative light units expressed as a % of Ctl ± SD; n = 6; **p < 0.01; ***p < 0.001; ****p < 0.0001. Western blots confirm down‐regulation of Eps8 using RNA interference. Equal loading was confirmed by HSC70. Numbers below the blots indicate the densitometry values measured using ImageJ normalized to HSC70 and expressed as a ratio to Ctl. [file PATH-243-37-s004.tif]

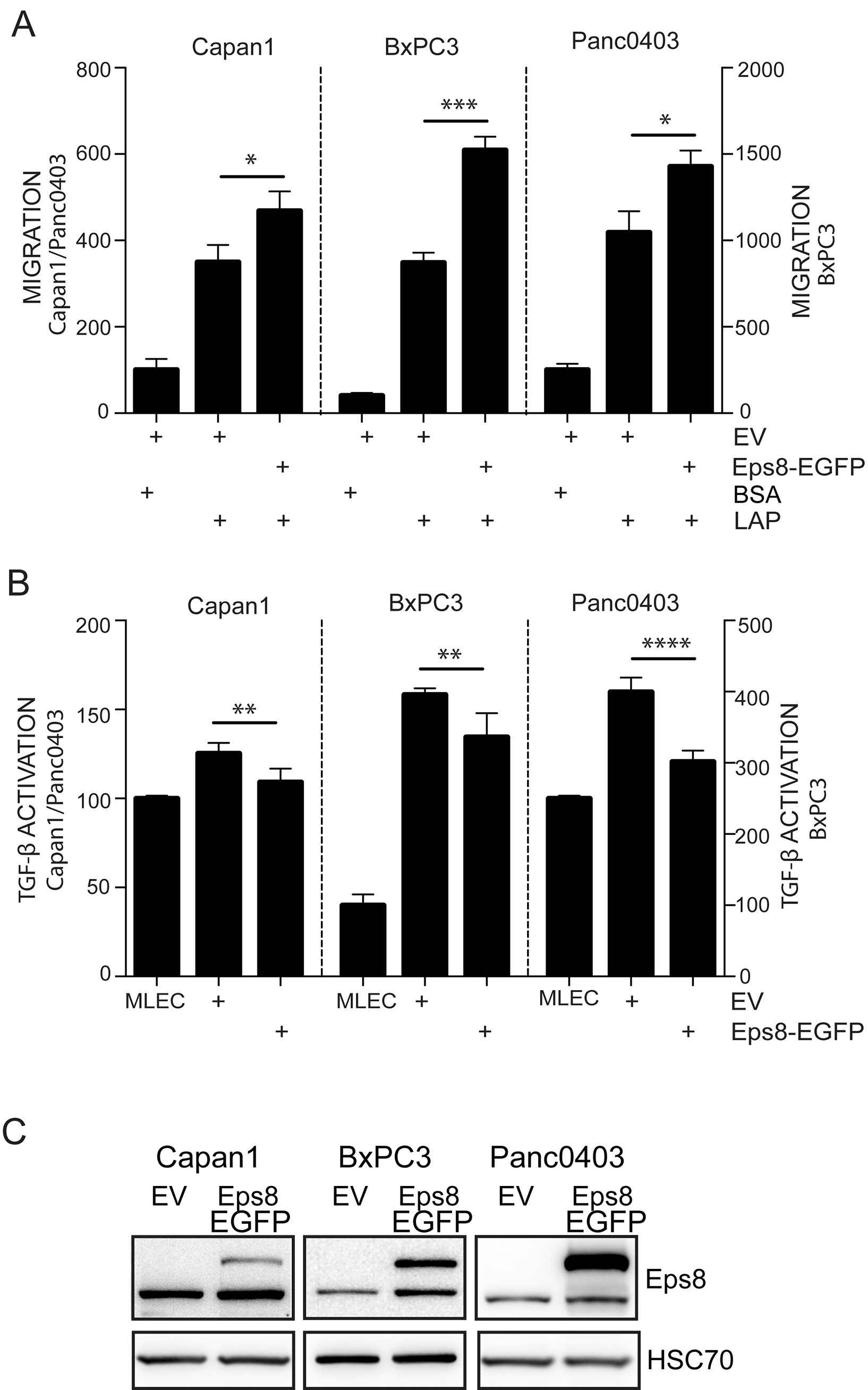

Supplement: Supplementary file 7 — Figure S5. Eps8 overexpression increases cell motility while it inhibits TGF‐β activation. (A) Capan1, BxPC3, and Panc0403 cells were transfected with empty vector (EV) or Eps8–EGFP 24 h before plating them into a Transwell® migration assay. Eps8 overexpression in all three cell lines significantly increased cell migration towards the αvβ6 ligand, LAP. Diagram represents the mean number of migrating cells per well expressed as a % of Ctl (BSA) ± SD (Capan1/Panc0403 plotted on left, BxPC3 plotted on right Y‐axis); n = 3; *p < 0.05; ***p < 0.001. (B) Capan1, BxPC3, and Panc0403 cells were transfected with empty vector (EV) or Eps8‐EGFP 24 h before plating them on top of MLEC cells. Eps8 overexpression significantly inhibited TGF‐β activation in all three cell lines. Diagram represents the mean relative light units expressed as a % of MLECs ± SD (Capan1/Panc0403 plotted on left, BxPC3 plotted on right Y‐axis); n = 6; **p < 0.01; ****p < 0.0001. (C) Eps8–EGFP expression was confirmed by western blotting. HSC70 was used as a loading control. [file PATH-243-37-s014.tif]

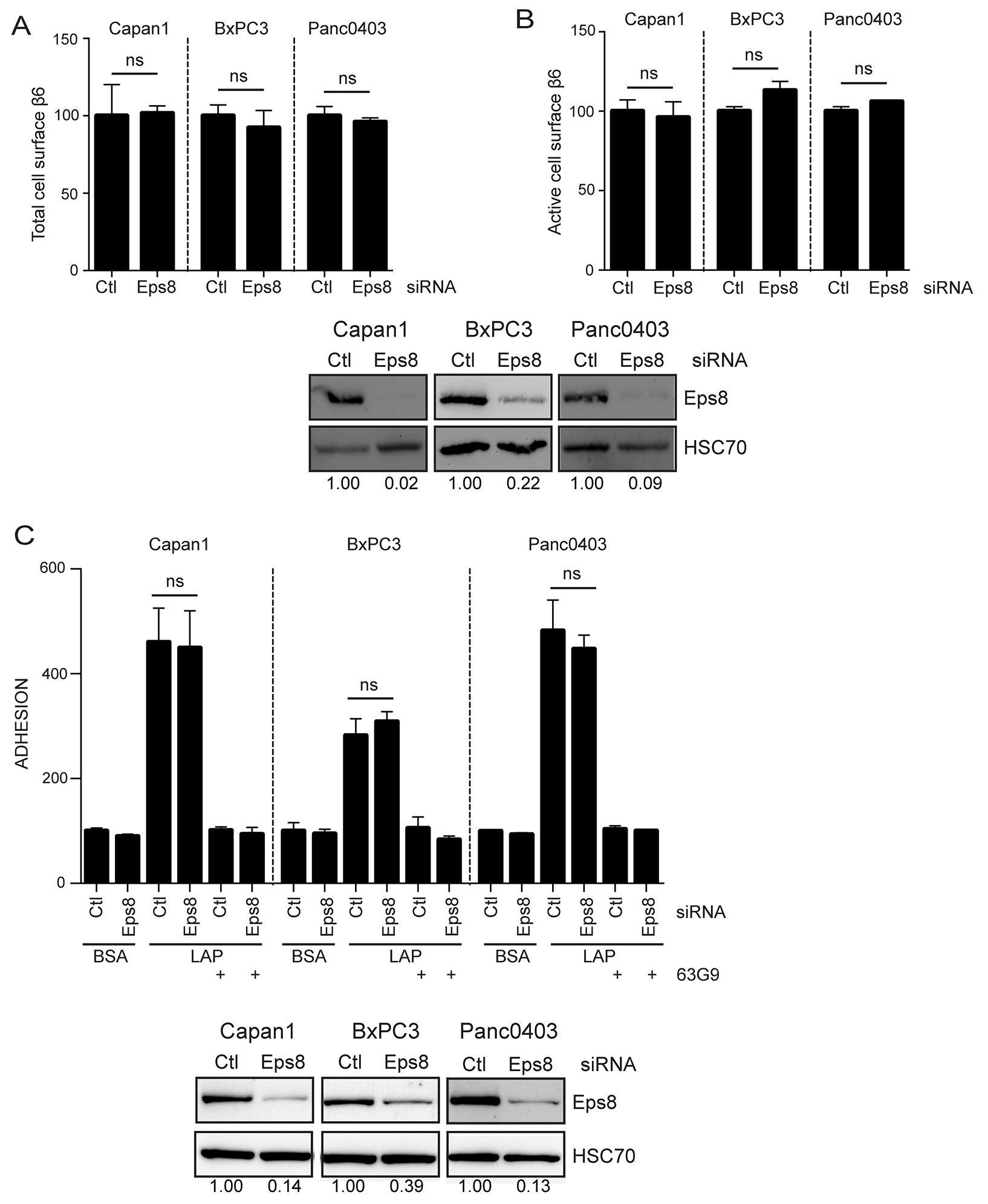

Supplement: Supplementary file 8 — Figure S6. Eps8 does not affect the cell surface levels of β6 integrin. Cells were transfected with non‐targeting (Ctl) or Eps8‐targeting siRNA, and the cell surface levels of total (A) or active (B) β6 integrin were measured by FACS analysis 48 h post‐transfection using either anti‐β6 (620 W) (A) or anti‐active β6 (6.2G2) antibodies (B). Diagrams represent the mean fluorescence intensity expressed as a % of Ctl ± SD; n = 3; ns = non‐significant. (C) Cells were transfected with either non‐targeting (Ctl) or Eps8‐targeting siRNA, and cell adhesion on LAP was measured 48 h post‐transfection. Eps8 down‐regulation had no effect on αvβ6‐specific adhesion of PDAC cells. Diagrams represent the absorbance at 540 nm expressed as a % of Ctl (BSA) ± SD; n = 4; ns = non‐significant. Western blots confirmed down‐regulation of Eps8 following siRNA transfection. Equal loading was confirmed by HSC70. Numbers below the blots indicate the densitometry values measured using ImageJ normalized to HSC70 and expressed as a ratio to Ctl. [file PATH-243-37-s006.tif]

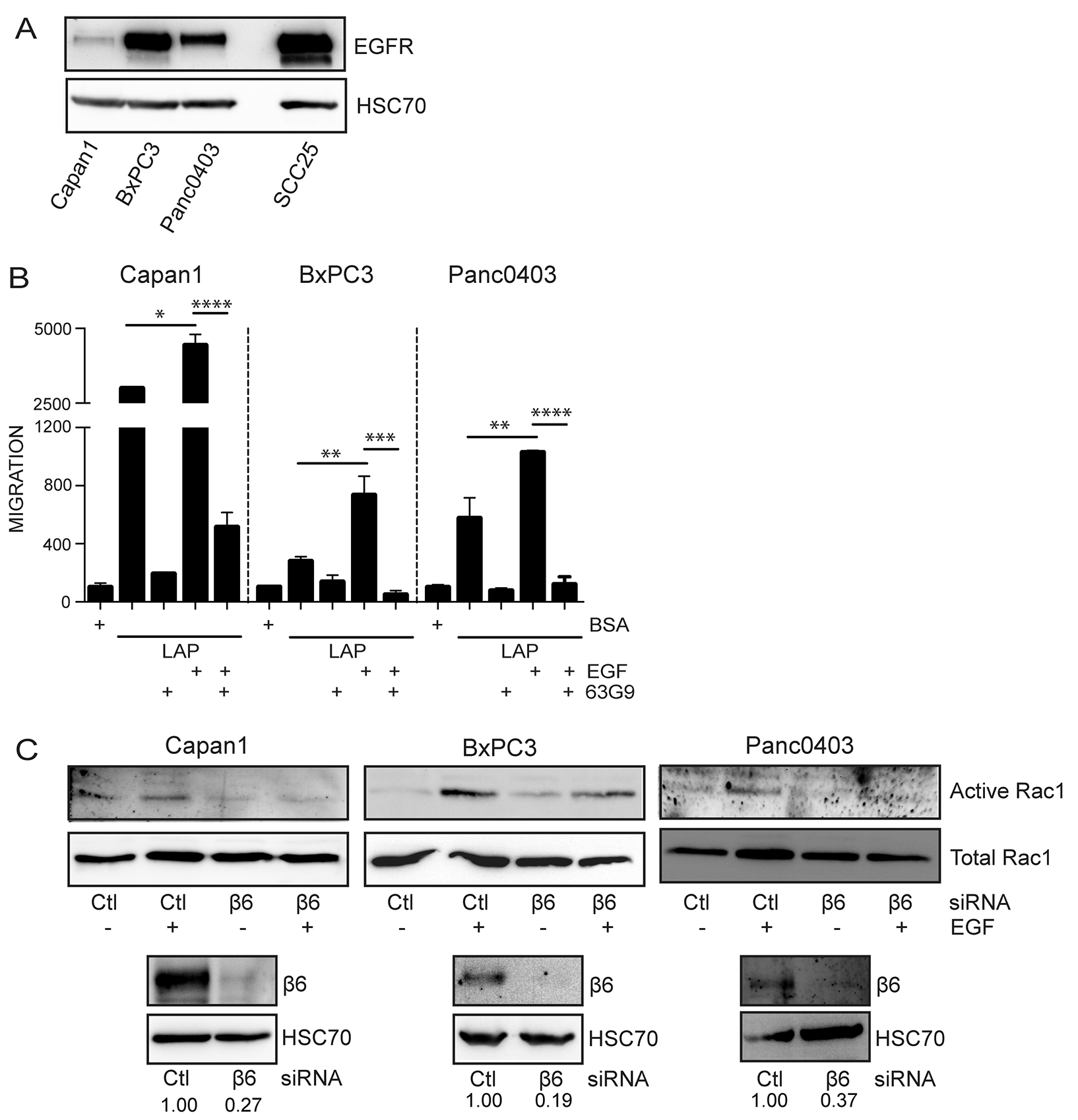

Supplement: Supplementary file 9 — Figure S7. EGF stimulation potentiates αvβ6 signalling and function. (A) Western blot showing expression of EGFR in the αvβ6‐positive Capan1, BxPC3, and Panc0403 cancer cells. The SCC25 oral squamous cell carcinoma cell line was used as a positive control. Equal loading was confirmed by HSC70. (B) Stimulation of Capan1, BxPC3, and Panc0403 cells with 20 ng/ml EGF induced a significant increase in migration levels towards the αvβ6 integrin ligand LAP. This EGF‐induced migration was completely inhibited by the αvβ6 blocking antibody 63G9, confirming that EGF‐induced migration of PDAC cells was αvβ6‐dependent. Diagram represents the mean number of migrating cells per well expressed as a % of BSA ± SD; n = 3; *p < 0.05; **p < 0.01; ***p < 0.001; ****p < 0.0001. (C) Stimulation of Capan1, BxPC3, and Panc0403 cells with 20 ng/ml EGF induced a significant activation of the small GTPase Rac1, as evidenced by a GST‐PAK1‐CRIB pull‐down assay. Knockdown of αvβ6 completely blocked EGF‐induced Rac1 activation in all cell lines, confirming αvβ6 dependency. β6 knockdown in the same lysates was confirmed on separate western blots. Equal loading was confirmed by HSC70. Numbers below the blots indicate the densitometry values measured using ImageJ normalized to HSC70 and expressed as a ratio to Ctl. [file PATH-243-37-s013.tif]

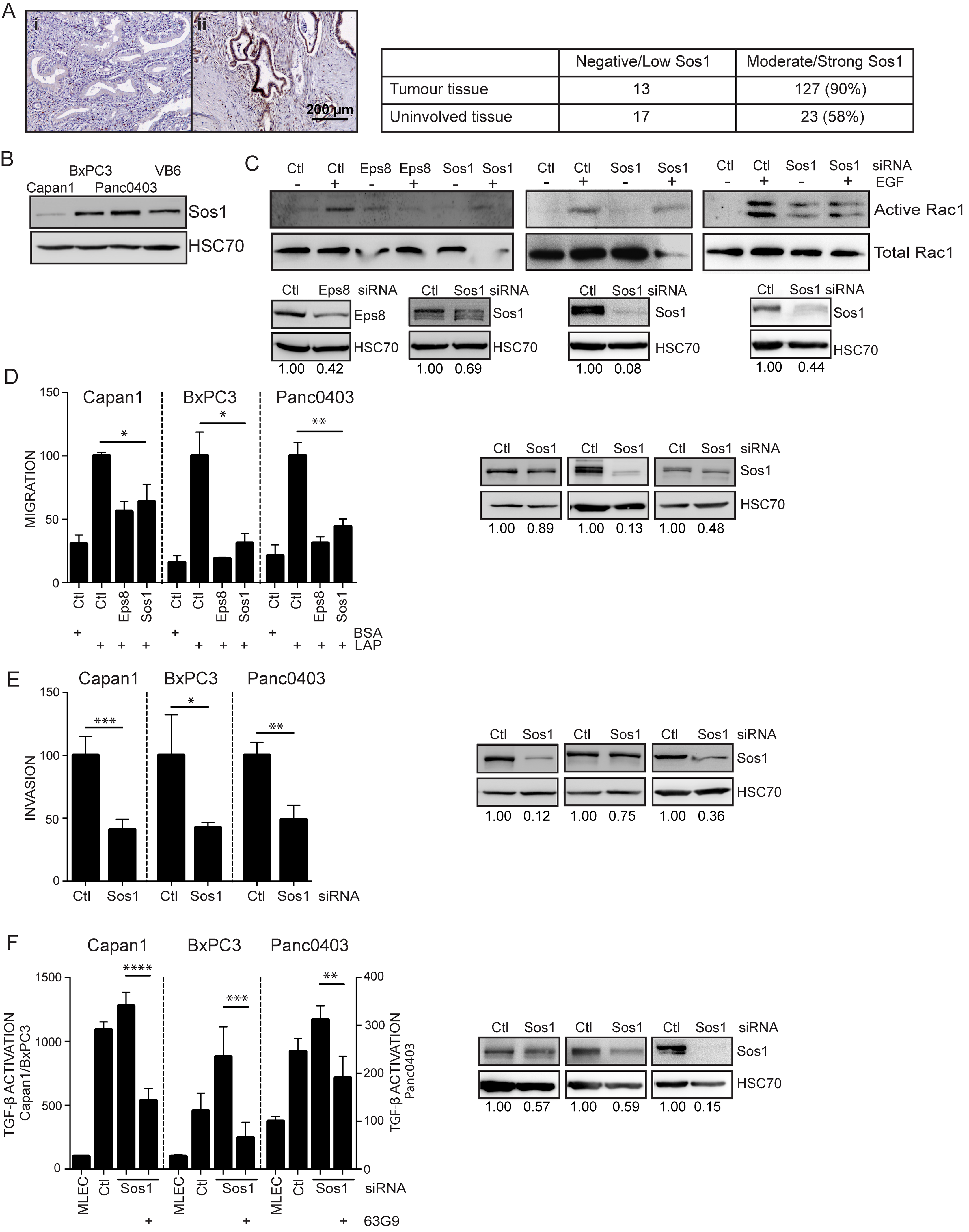

Supplement: Supplementary file 10 — Figure S8. Sos1 is overexpressed in PDAC and promotes motility but inhibits TGF‐β activation. (A) Representative image of the immunohistochemical staining of Sos1 in PDAC (ii; tumour) and surrounding (i; uninvolved) tissue. The table on the right shows the staining intensity using the QuickScore method. (B) Western blot showing Sos1 expression in three αvβ6‐positive PDAC cell lines. The human oral squamous cell carcinoma cell line VB6 was used as a positive control. Equal loading was confirmed by HSC70. (C) Results of a GST pull‐down assay using GST‐PAK1‐CRIB‐coated Sepharose beads showing that Sos1 knockdown completely inhibits EGF‐induced Rac1 activation compared with cells transfected with non‐targeting (Ctl) siRNA in Capan1, BxPC3, and Panc0403 cells. The western blot for Capan1 cells originated from the same experiment presented in Figure 3A. Eps8 and Sos1 knockdown in the same lysates was confirmed on separate western blots. Equal loading was confirmed by HSC70. Numbers below the blots indicate the densitometry values measured using ImageJ normalized to HSC70 and are expressed as a ratio to Ctl. (D) Sos1 down‐regulation by RNA interference significantly inhibits Transwell® migration of Capan1, BxPC3, and Panc0403 cells towards the αvβ6 integrin ligand LAP compared with non‐targeting (Ctl) siRNA. Diagram represents the mean number of migrated cells per well expressed as a % of Ctl (LAP) ± SD; n = 3; *p < 0.05; **p < 0.01. (E) Invasion of Capan1, BxPC3, and Panc0403 cells through Matrigel‐coated Transwells® was significantly inhibited by down‐regulation of Sos1 using RNA interference. Diagram represents the mean number of invaded cells per well expressed as a % of Ctl ± SD; n = 4; *p < 0.05; **p < 0.01; ***p < 0.001. (F) Sos1 knockdown induced activation of TGF‐β1 compared with non‐targeting (Ctl) siRNA‐transfected cells in an MLEC TGF‐β activation assay. Sos1‐induced TGF‐β activation was inhibited by the αvβ6 blocking antibody 63G9. Diagram represents the mean [file PATH-243-37-s012.tif]

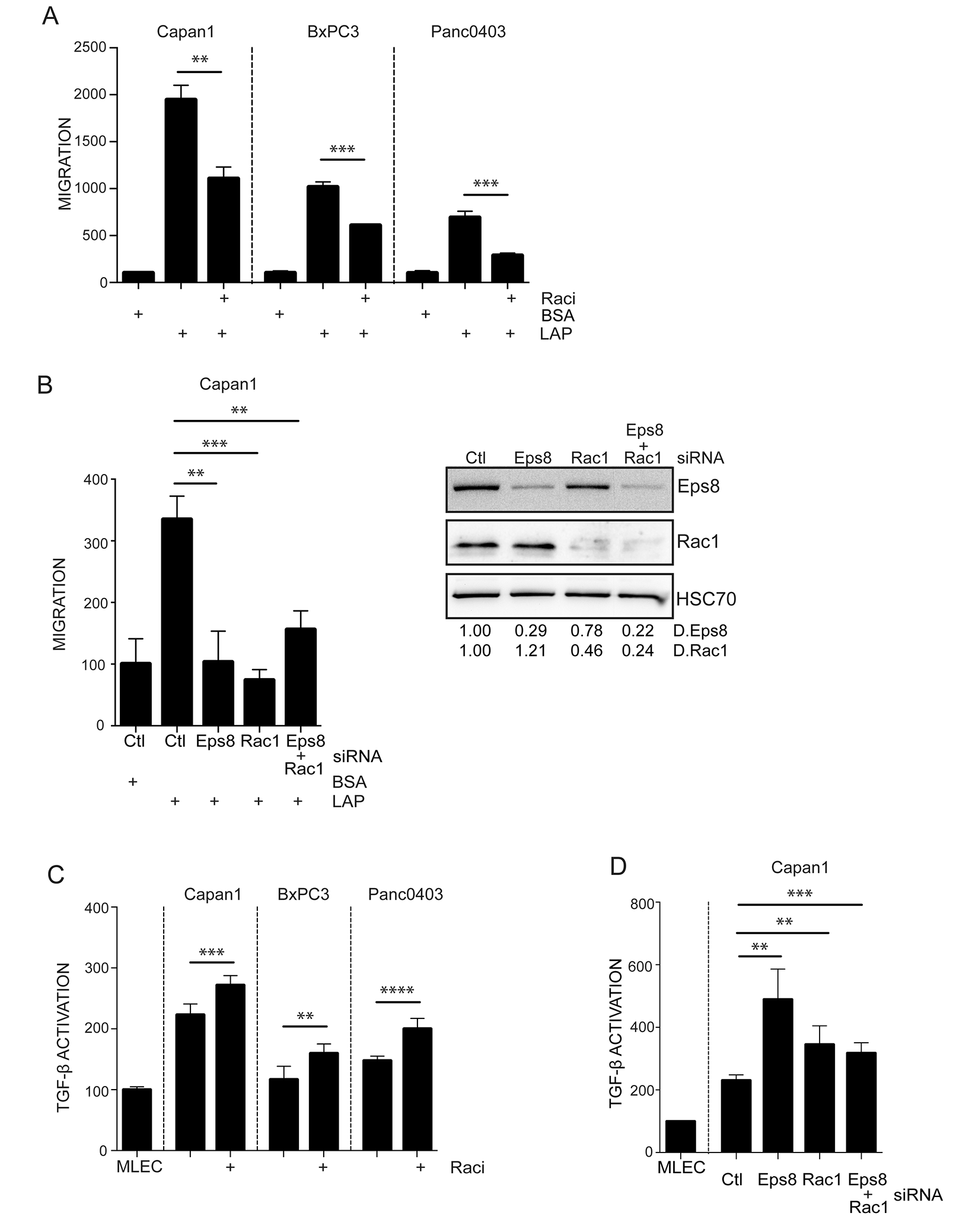

Supplement: Supplementary file 11 — Figure S9. Eps8 and Rac1 regulate cell motility and TGF‐β activation in the same pathway. (A) Transwell® migration of Capan1, BxPC3, and Panc0403 cells towards LAP is inhibited by overnight pretreatment with 50 μm of the Rac1 inhibitor, NSC23766 (Raci). Diagram represents the mean number of migrated cells per well expressed as a % of Ctl (BSA) ± SD; n = 3; **p < 0.01; ***p < 0.001. (B) Transwell® migration of Capan1 cells towards LAP was inhibited to the same extent by down‐regulation of Eps8 or Rac1 and simultaneous down‐regulation of both proteins. Diagram represents the mean number of migrated cells per well expressed as a % of Ctl (BSA) ± SD; n = 3; **p < 0.01; ***p < 0.001. Western blots confirm down‐regulation of Eps8 and Rac1. Equal loading was confirmed by HSC70. Numbers below the blots indicate the densitometry values for Eps8 (D˙Eps8) and Rac1 (D.Rac1) knockdown measured using ImageJ normalized to HSC70 and expressed as a ratio to Ctl. (C) Capan1, BxPC3, and Panc0403 cells were pretreated overnight by 50 μm of the Rac1 inhibitor NSC23766 (Raci), after which they were plated on top of MLEC cells in the absence of the inhibitor. Rac1 inhibition significantly increased TGF‐β activation in all three cell lines. Diagram represents the mean relative light units expressed as a % of MLECs ± SD; n = 6; **p < 0.01; ***p < 0.001; ****p < 0.0001. (D) Eps8, Rac1 or Eps8 and Rac1 siRNA induced significantly increased TGF‐β activation in Capan1 cells. Diagram represents the mean relative light units expressed as a % of MLECs ± SD; n = 6; **p < 0.01; ***p < 0.001. Western blots in B confirmed down‐regulation of Eps8 and Rac1 using RNA interference. [file PATH-243-37-s017.tif]

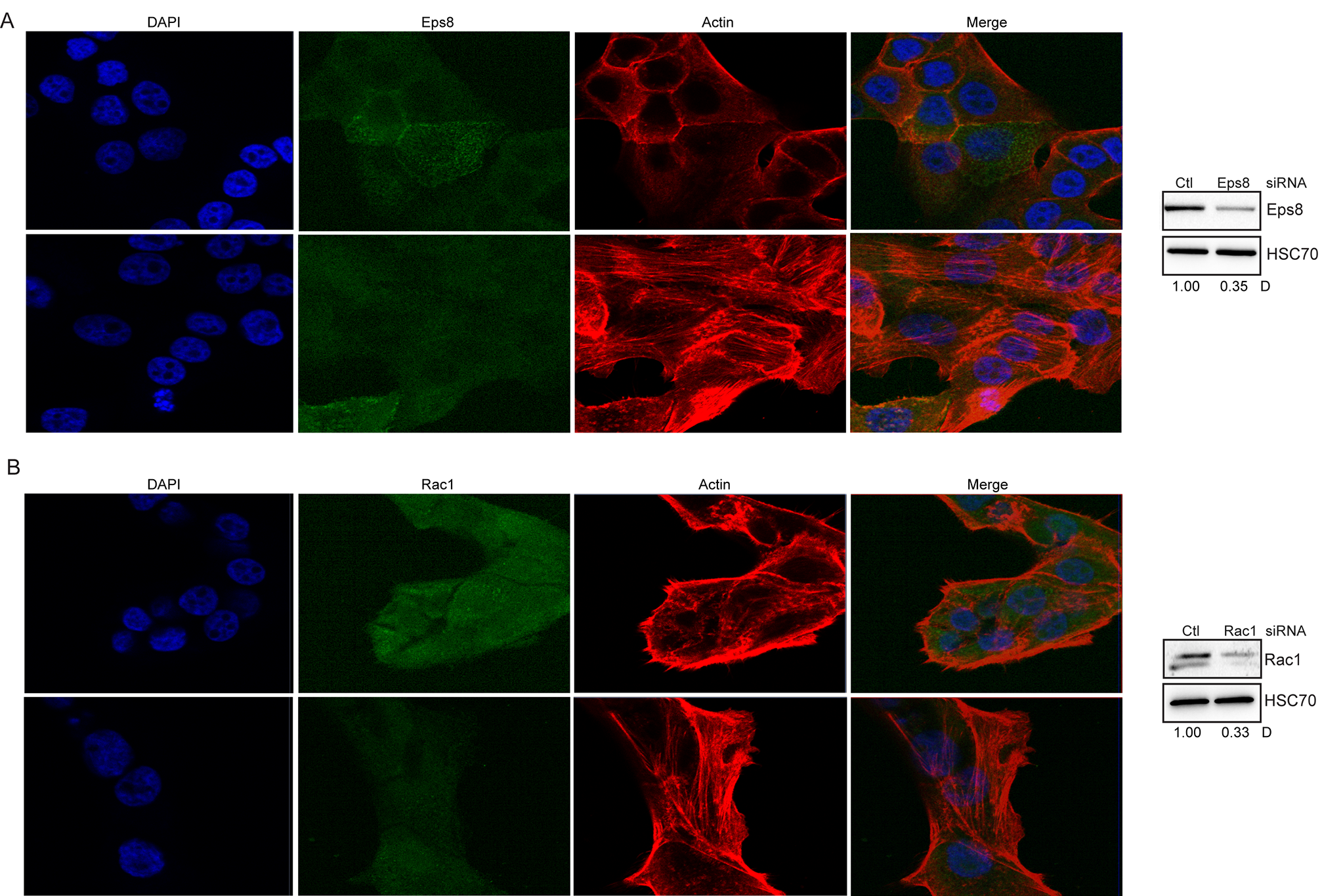

Supplement: Supplementary file 12 — Figure S10. Stress‐fibre formation was reduced in cells with incomplete knockdown of Eps8 and Rac1. Capan1 cells were transfected with Eps8 or Rac1 siRNA, plated on 0.5 μg/ml LAP‐coated coverslips, and after an overnight incubation stained with phalloidin‐FITC (red) to visualize stress‐fibre formation or anti‐Eps8 or Rac1 antibodies (green) to detect the level of knockdown. DAPI (blue) was used as a nuclear counterstain. Cells with absent or very low levels of Eps8 (A, bottom panels) and Rac1 (B, bottom panels) expression showed increased stress‐fibre formation, whereas cells in which Eps8 (A, top panels) and Rac1 (B, top panels) down‐regulation was incomplete did not produce stress fibres. Images were captured at the same microscope setting and exposure was uniformly enhanced across images to aid better visibility. Representative images are shown. Western blots confirm down‐regulation of Eps8 or Rac1 following siRNA transfection. Equal loading was confirmed by HSC70. Numbers below the blots indicate the densitometry values measured using ImageJ normalized to HSC70 and expressed as a ratio to Ctl. [file PATH-243-37-s009.tif]

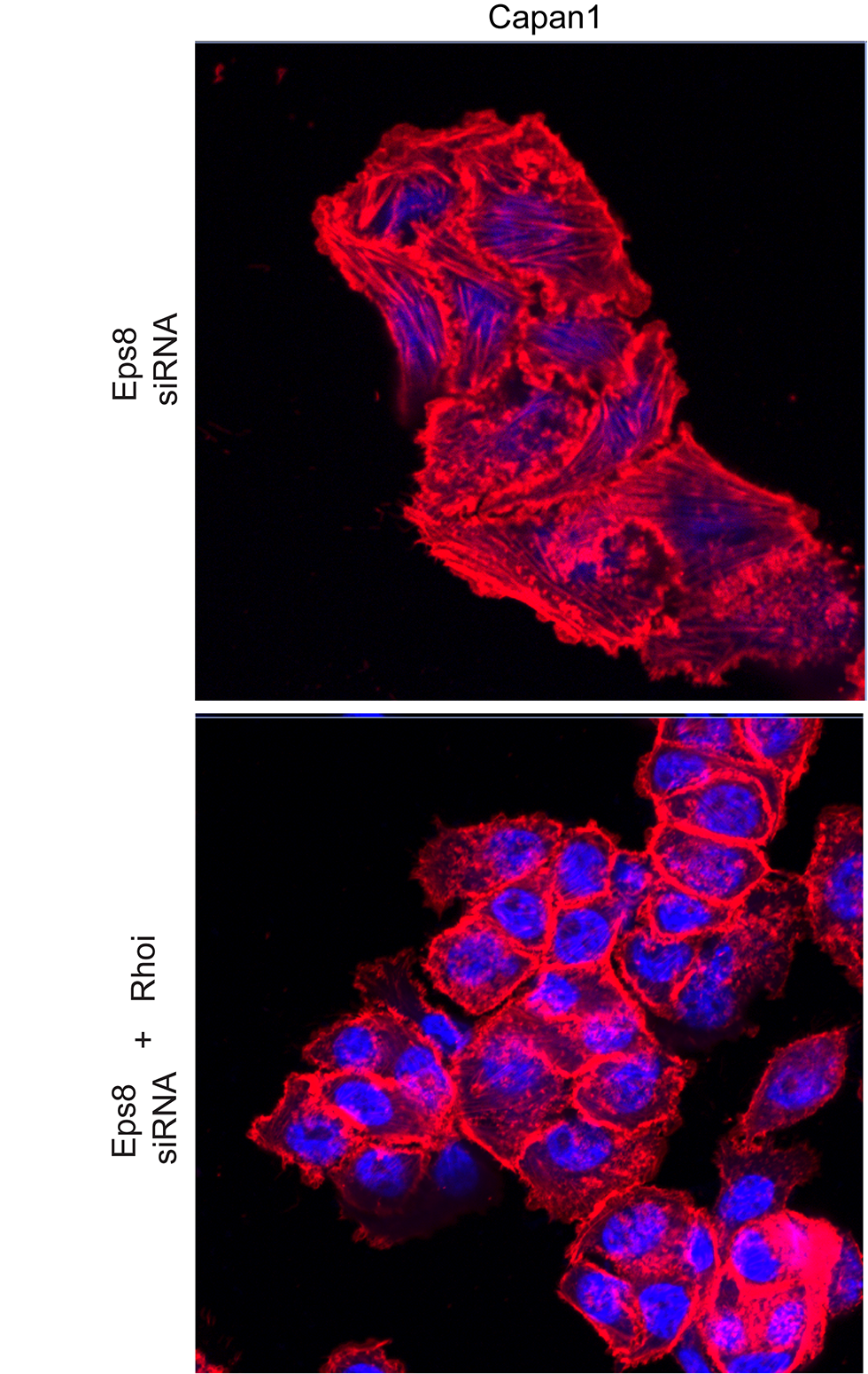

Supplement: Supplementary file 13 — Figure S11. The cell‐permeable Rho inhibitor CT04 inhibits stress‐fibre formation in Eps8 knockdown cells. Capan1 cells transfected with Eps8 siRNA were plated on 0.5 μg/ml LAP‐coated coverslips in the presence of 1% serum until they had fully adhered and spread. Medium on the cells was changed to serum‐free medium and cells were incubated in the absence (top panel) or presence (bottom panel) of 0.5 μg/ml CT04 Rho inhibitor. After overnight incubation, cells were fixed and stained with phalloidin‐FITC (red) to visualize stress‐fibre formation. DAPI (blue) was used as a nuclear counterstain. Exposure was uniformly enhanced across images to aid better visibility. A representative image is shown. [file PATH-243-37-s016.tif]

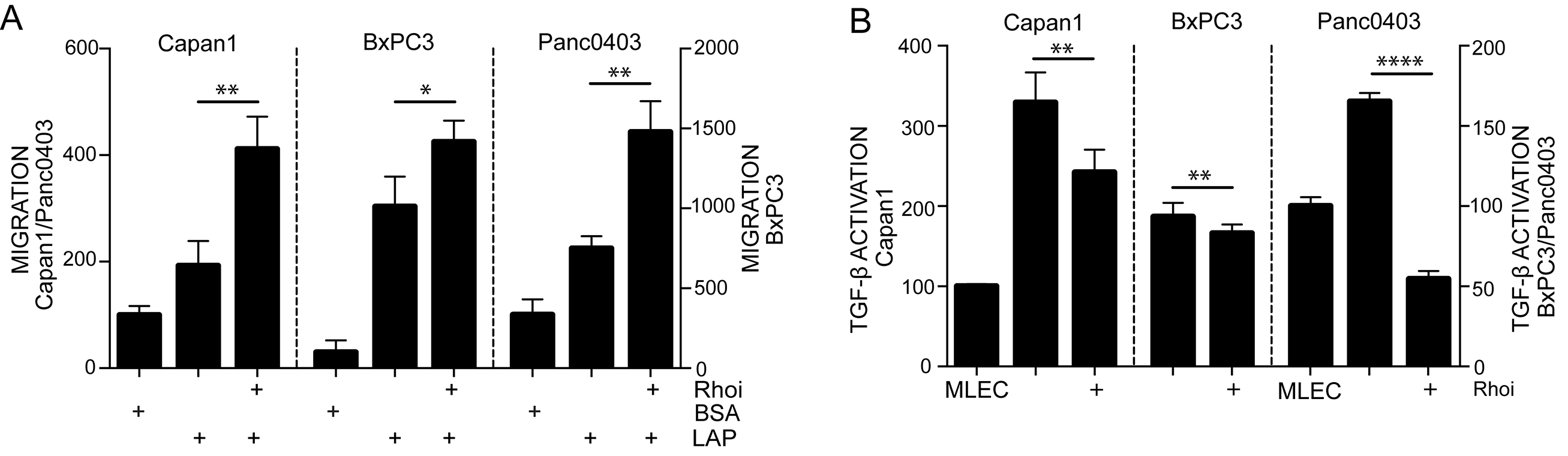

Supplement: Supplementary file 14 — Figure S12. Rho inhibition induces cell motility and inhibits TGF‐β activation. (A) Capan1, BxPC3, and Panc0403 cells were pretreated with 0.5 μg/ml of the CT04 Rho inhibitor (Rhoi) before plating them into a Transwell® migration assay. Migration towards LAP of all three cell lines was significantly increased by Rho inhibition. Diagram represents the mean number of migrated cells per well expressed as a % of Ctl (BSA) ± SD (Capan1/Panc0403 plotted on left, BxPC3 plotted on right Y‐axis); n = 3; *p < 0.05; **p < 0.01. (B) Capan1, BxPC3, and Panc0403 cells were pretreated by 0.5 μg/ml of the CT04 Rho inhibitor (Rhoi), after which they were plated on top of MLEC cells in the absence of the inhibitor. Rho inhibition significantly inhibited TGF‐β activation in all three cell lines. Diagram represents the mean relative light units expressed as a % of MLECs ± SD (Capan1 plotted on left, BxPC3/Panc0403 plotted on right Y‐axis); n = 6; **p < 0.01; ****p < 0.0001. [file PATH-243-37-s011.tif]

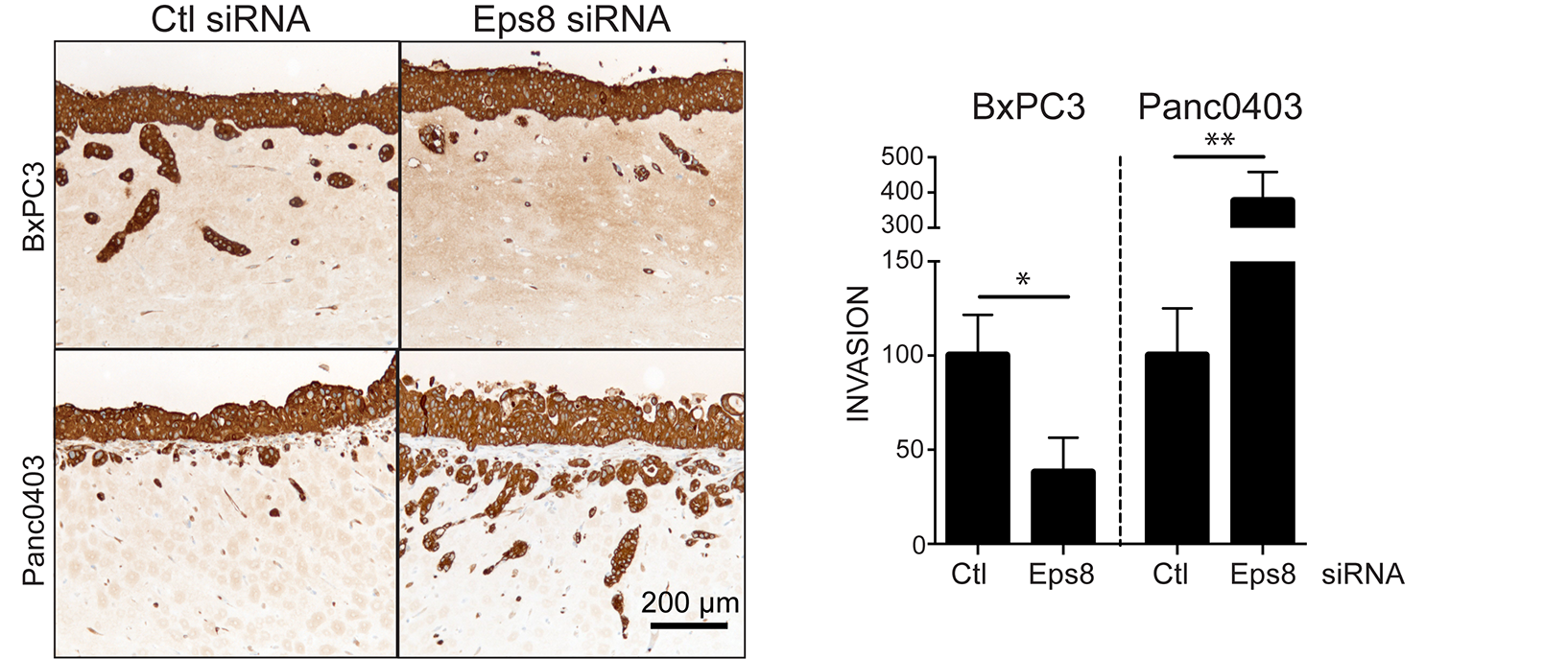

Supplement: Supplementary file 15 — Figure S13. BxPC3 and Panc0403 cells invade differently in the presence of primary pancreatic stellate cells. BxPC3 and Panc0403 cells were transfected with either non‐targeting (Ctl) or Eps8‐targeting siRNA, and organotypic invasion assays were performed in the presence of primary pancreatic stellate cells over a period of 12 days. While invasion of BxPC3 cells was significantly inhibited by down‐regulation of Eps8 compared with Ctl cells, invasion of Panc0403 cells was significantly increased. A representative image of cytokeratin‐stained sections is shown. Diagram shows the mean invasion depth of three independent sections analysed by ImageJ software expressed as a % of Ctl ± SD; n = 3; *p < 0.05; **p < 0.01. [file PATH-243-37-s008.tif]

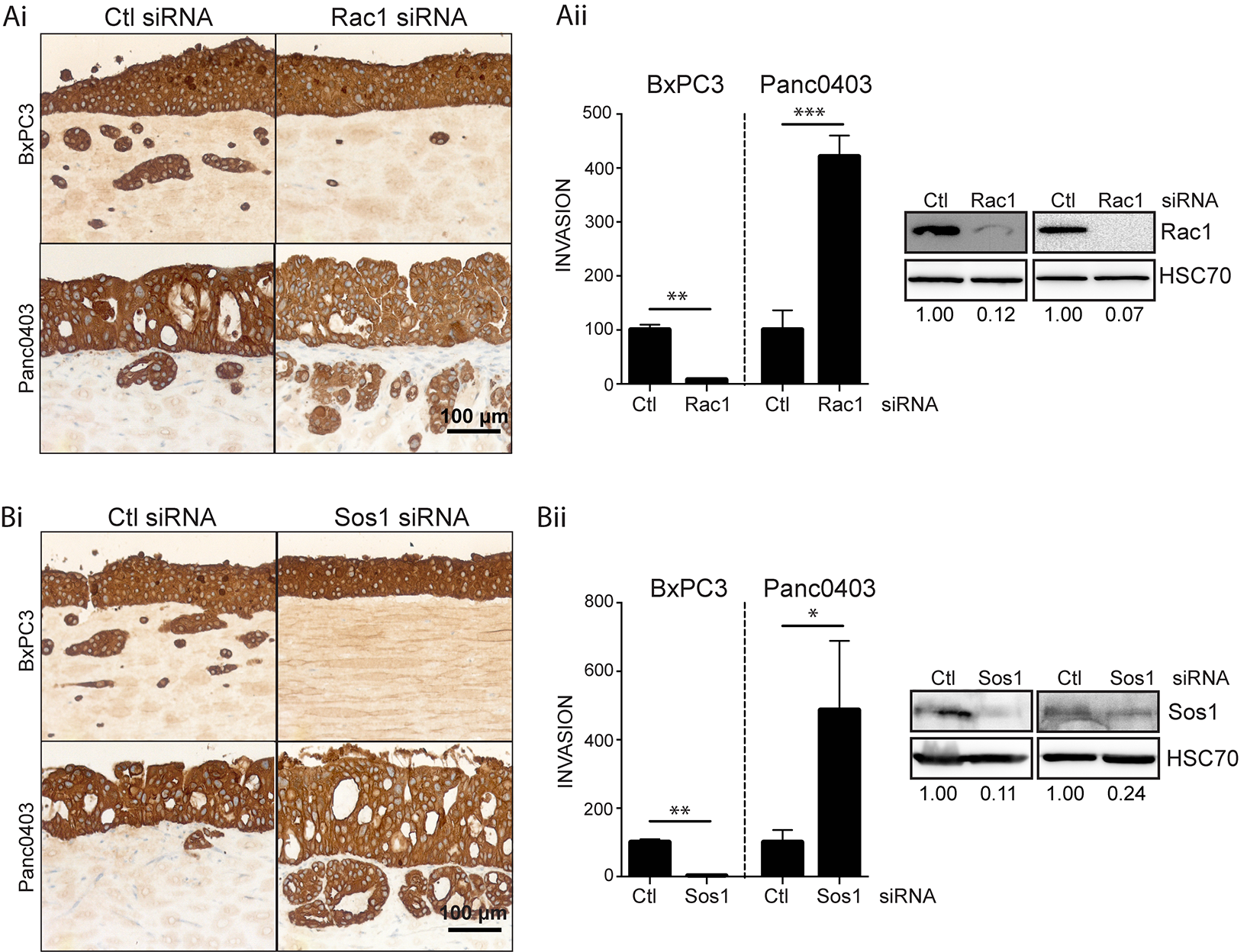

Supplement: Supplementary file 16 — Figure S14. Rac1 and Sos1 inhibit the invasion of Panc0403 but not that of BxPC3 cells. (Ai) BxPC3 and Panc0403 cells were transfected with either non‐targeting (Ctl) or Rac1‐targeting siRNA, and organotypic invasion assays were performed in the presence of HFFF2 fibroblasts over a period of 12 days. While down‐regulation of Rac1 inhibited the invasion of BxPC3 cells compared with Ctl cells, it induced a significant level of invasion in Panc0403 cells. A representative image of a cytokeratin‐stained section is shown. (Aii) Diagrams show the mean invasion depth of three independent sections analysed by ImageJ software expressed as % of Ctl ± SD; n = 3; **p < 0.01; ***p < 0.001. Western blots confirmed down‐regulation of Rac1. Equal loading was confirmed by HSC70. (Bi) BxPC3 and Panc0403 cells were transfected with either non‐targeting (Ctl) or Sos1‐targeting siRNA, and organotypic invasion assays were performed in the presence of HFFF2 fibroblasts over a period of 12 days. While down‐regulation of Sos1 inhibited the invasion of BxPC3 cells compared with Ctl cells, it induced a significant level of invasion in Panc0403 cells. A representative image of a cytokeratin‐stained section is shown. (Bii) Diagrams show the mean invasion depth of three independent sections analysed by ImageJ software expressed as a % of Ctl ± SD; n = 3; *p < 0.05; **p < 0.01. Western blots confirmed down‐regulation of Sos1. Equal loading was confirmed by HSC70. Numbers below the blots in Aii and Bii indicate the densitometry values measured using ImageJ normalized to HSC70 and expressed as a ratio to Ctl. [file PATH-243-37-s002.tif]
