# Supplementary material for: Pro‐migratory and TGF‐β‐activating functions of αvβ6 integrin in pancreatic cancer are differentially regulated via an Eps8‐dependent GTPase switch
Source: J Pathol. 2017 Aug 7;243(1):37–50. doi: 10.1002/path.4923 (PMC5601247; doi:10.1002/path.4923)
Supplement: Supplementary file 17 — Table S1. Antibodies used in the study [file PATH-243-37-s005.docx]

**Table** **S1.** Antibodies used in the study

| **Primary antibody** | **Species** | **Catalogue/clone number** | **Supplier** | **IHC/IF dilution** | **WB/FACS/blocking concentration** |
| --- | --- | --- | --- | --- | --- |
| HSC70 | Mouse | sc-7298 / B-6 | Santa Cruz Biotechnology  (Dallas, TX, USA) | N/A | 1:10 000 |
| 6.3G9 (β6) Blocking | Mouse | [26] / 25-3-1 | Biogen Idec  (Cambridge, MA, USA) | N/A | 10 μg/ml for blocking |
| 6.20w (β6) | Rat | N/A / N/A | In-house | N/A | 1:500 / 20 μg/ml / N/A |
| 62G2 (β6) | Mouse | [26] / 87-1 | Biogen Idec  (Cambridge, MA, USA) | 2 μg/ml / N/A | N/A / 10 μg/ml / N/A |
| αV | Rabbit | 4711 / N/A | Cell Signaling Technology  (Danvers, MA, USA) | N/A | 1:1000 / N/A / N/A |
| Eps8 | Mouse | 610144 / 15 | BD Biosciences  (Franklin Lakes, NJ, USA) | 1:400 / 1:100 | 1:2 000 / N/A / N/A |
| Rac1 | Mouse | 05-389 / 23A8 | Millipore  (Watford, UK) | N/A | 1:1 000 / N/A / N/A |
| **Primary antibody** | **Species** | **Catalogue/clone number** | **Supplier** | **IHC/IF dilution** | **WB/FACS/blocking concentration** |
| Rac1 | Mouse | ARC03 / N/A | Cytoskeleton  (Denver, CO, USA) | N/A / 25 μg/ml | N/A |
| Sos1 | Rabbit | sc-17793 / A-9 | Santa Cruz Biotechnology  (Dallas, TX, USA) | 1:400 / N/A | 1:1 000 / N/A / N/A |
| αSMA | Mouse | M0851 / 1A4 | Dako  (Ely, UK) | 1:250 / 1:100 | N/A |
| αSMA | Mouse | A2547 / 1A4 | Sigma-Aldrich  (Gillingham, UK) | N/A | 1:1 000 / N/A / N/A |
| Cytokeratin | Mouse | IR053 / AE1/AE3 | Dako  (Ely, UK) | 1:100 / N/A | N/A |
| Cytokeratin | Rabbit | Z0622 / N/A | Dako  (Ely, UK) | N/A / 1:500 | N/A |
| EGFR | Goat | AF231 / N/A | R&D Systems  (Minneapolis, MN, USA) | N/A | 1:1000 / N/A / N/A |

N/A = not applicable.
