# Supplementary material for: Pro‐migratory and TGF‐β‐activating functions of αvβ6 integrin in pancreatic cancer are differentially regulated via an Eps8‐dependent GTPase switch
Source: J Pathol. 2017 Aug 7;243(1):37–50. doi: 10.1002/path.4923 (PMC5601247; doi:10.1002/path.4923)
Supplement: Supplementary file 18 — Table S2. siRNAs used in the study [file PATH-243-37-s015.docx]

**Table S2.** siRNAs used in the study

| **Species_target** | **Target sequence** | **Supplier** | **Catalogue number** | **Concentration (nm)** |
| --- | --- | --- | --- | --- |
| **Hs_ITGB6** | ON-TARGETplus SMARTpool | Dharmacon/  Fermentas | L-008012-00-0020 | 100 |
| **EPS8** | GGCCCTTTATGAACAAAGG | Ambion/Life Technologies | AM16706 | 30 |
| **Hs_EPS8_1** | TCGGTTCTAAAGGATGATATT | Qiagen | SI00380737 | 30 |
| **Hs_EPS8_2** | TTGGATATTGTGAGACCTCCA | Qiagen | SI00380744 | 30 |
| **Hs_EPS8_3** | CAGGTGGATGTTAGAAGTCGA | Qiagen | SI00380751 | 30 |
| **Hs_RAC1** | CTACTGTCTTTGACAATTA | Ambion/Life Technologies | 4390825 | 30 |
| **Hs_SOS1** | ON-TARGETplus SMARTpool | Dharmacon/  Fermentas | L-005194-00-0005 | 30 |
